# Tumor Susceptibility Gene 101 (TSG101) Contributes to Virion Formation of Porcine Reproductive and Respiratory Syndrome Virus via Interaction with the Nucleocapsid (N) Protein along with the Early Secretory Pathway

**DOI:** 10.1128/jvi.00005-22

**Published:** 2022-03-23

**Authors:** Longxiang Zhang, Rui Li, Rui Geng, Lei Wang, Xin-xin Chen, Songlin Qiao, Gaiping Zhang

**Affiliations:** a College of Veterinary Medicine, Northwest A&F University, Yangling, Shaanxi, China; b Key Laboratory of Animal Immunology of the Ministry of Agriculture, Henan Provincial Key Laboratory of Animal Immunology, Henan Academy of Agricultural Sciences, Zhengzhou, Henan, China; Instituto de Biotecnologia/UNAM

**Keywords:** PRRSV, ESCRT, TSG101, N protein, assembly

## Abstract

Porcine reproductive and respiratory syndrome virus (PRRSV) has caused huge economic losses to global swine industry. As an intracellular obligate pathogen, PRRSV exploits host cellular machinery to establish infection. The endocytic sorting complex required for transport (ESCRT) system has been shown to participate in different life cycle stages of multiple viruses. In the present study, a systematic small interference RNA screening assay identified that certain ESCRT components contributed to PRRSV infection. Among them, tumor susceptibility gene 101 (TSG101) was demonstrated to be important for PRRSV infection by knockdown and overexpression assays. TSG101 was further revealed to be involved in virion formation rather than viral attachment, internalization, RNA replication and nucleocapsid (N) protein translation within the first round of PRRSV life cycle. In detail, TSG101 was determined to specially interact with PRRSV N protein and take effect on its subcellular localization along with the early secretory pathway. Taken together, these results provide evidence that TSG101 is a proviral cellular factor for PRRSV assembly, which will be a promising target to interfere with the viral infection.

**IMPORTANCE** PRRSV infection results in a serious swine disease affecting pig farming in the world. However, efficient prevention and control of PRRSV is hindered by its complicated infection process. Until now, our understanding of PRRSV assembly during infection is especially limited. Here, we identified that TSG101, an ESCRT-I subunit, facilitated virion formation of PRRSV via interaction with the viral N protein along with the early secretory pathway. Our work actually expands the knowledge of PRRSV infection and provides a novel therapeutic target for prevention and control of the virus.

## INTRODUCTION

Porcine reproductive and respiratory syndrome (PRRS) is a highly contagious swine disease that causes reproductive failures in sows of the late-term gestation and acute respiratory distress in pigs of all ages (1, 2). PRRS has led to tremendous economic losses to swine industry worldwide since it emerged in the United States and Europe almost simultaneously in the late 1980s (3, 4). Annual economic losses in the United States due to PRRS is estimated up to $664 million (5). Its causative agent, PRRS virus (PRRSV), is an enveloped, nonsegmented, single-stranded positive-sense RNA virus and belongs to the order *Nidovirales*, family *Arteriviridae*, and genus *Betaarterivirus* (6). The genome of PRRSV is approximately 15 kb in length, and encodes two long polyproteins (pp1a and pp1ab) and eight structural proteins (glycoprotein 2 [GP2], envelope [E] protein, GP3, GP4, GP5, GP5a, matrix [M] protein, and nucleocapsid [N] protein) (7, 8). pp1a and pp1ab are further cleaved into at least 16 nonstructural proteins (Nsps), including Nsp1α, Nsp1β, Nsp2TF, Nsp2N, Nsp2-Nsp6, Nsp7α, Nsp7β, and Nsp8-Nsp12 (9).

Infection by PRRSV exhibits a highly restricted tropism for target cells, including porcine alveolar macrophages (PAMs), African green monkey kidney epithelial cell line MA-104, and its derivative MARC-145 cells (10, 11). The life cycle of PRRSV can be divided into attachment, internalization, replication, translation, assembly, and release (12). As an intracellular obligate pathogen, PRRSV exploits host cellular machinery to establish infection. PRRSV infection is initiated by attachment to host cell receptors/factors on the plasma membrane (13). Upon internalization, PRRSV utilizes the host machinery to finish its translation, transcription, and replication (14). Subsequently, PRRSV nucleocapsid buds into the smooth endoplasmic reticulum (ER) and/or Golgi apparatus (GA) to form enveloped virions. Finally, PRRSV virions are released via exocytosis for a new infectious cycle (15–17). Although certain ones have been identified, there is still a lack of research on host cellular factors involved in PRRSV infection, especially during assembly (18–21).

The endocytic sorting complex required for transport (ESCRT) system is a conserved and versatile molecular machinery comprising of five functionally distinct subcomplexes, namely, ESCRT-0, -I, -II, and -III, as well as several accessory proteins (22–24). It contains more than 30 proteins in mammalian cells. These proteins function in manipulating membrane bilayers in a variety of essential cellular physiological and metabolic processes, such as multivesicular body (MVB) formation, membrane abscission in cytokinesis, exosome and microvesicle biogenesis, nuclear envelope reconstruction, plasma membrane repair, neuron pruning, autophagosome closure, and lysosome repair (23–28). In addition, the ESCRT machinery is reported to be utilized by viruses from diverse families to support their infections at various stages (29–31).

ESCRT-I is a heterotetramer consisting of a 1:1:1:1 complex of tumor susceptibility gene 101 (TSG101), vacuolar protein sorting 28 (VPS28), VPS37A/B/C/D, and MVB12A/B in eukaryotic cells. As a crucial member, TSG101 has been reported to participate in efficient internalization of some viruses, including Lassa virus (32), rotavirus (33) and Crimean-Congo hemorrhagic fever virus (34), in replication of classical swine fever virus (35), and in assembly and release of various viruses (36–41). A previous study has shown that depletion of TSG101 inhibits PRRSV infection, but the detailed mechanisms remain to be fully elucidated (42).

In this study, we performed a systematic small interference RNA (siRNA) screening assay and identified certain ESCRT components important for PRRSV infection. Among them, TSG101 was chosen for further investigation on its involvement in PRRSV infection through multiple approaches.

## RESULTS

### Identification of the ESCRT components required for PRRSV infection.

**FIG 1:**
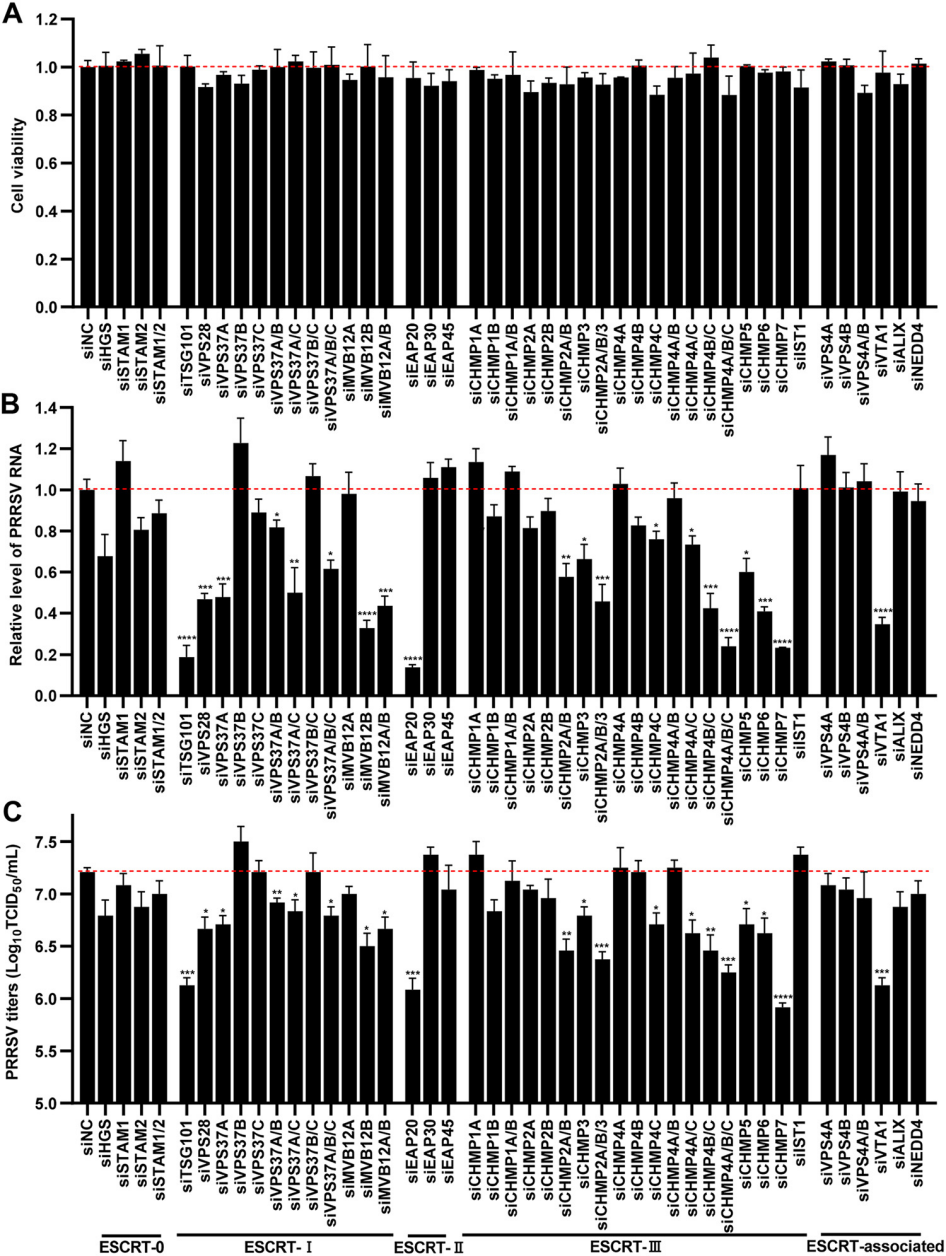
Identification of the ESCRT proteins required for PRRSV infection. (A) Cytotoxicities of the indicated siRNAs. The MARC-145 cells grown in 96-well plates were transfected with the indicated siRNAs, and the cell viabilities were evaluated by performing a CellTiter 96 AQueous One solution cell proliferation assay. The cell viability in siNC-transfected cells was set to 1.0. (B and C) Identification of ESCRT proteins required for PRRSV infection. The siRNA-transfected cells were inoculated with PRRSV at an MOI of 0.5 for 24 h. (B) PRRSV RNA abundance was determined by RT-qPCR. (C) The progeny PRRSV titers in the supernatant were measured by assessing TCID_50_. Data represent means ± the SEM from three independent experiments. Statistical analysis was carried out using one-way ANOVA. *, *P* < 0.05; **, *P* < 0.01; ***, *P* < 0.001; ****, *P* < 0.0001.

To investigate which ESCRT components were required for PRRSV infection, we performed a systematic siRNA screening assay. Initially, we designed three pairs of siRNA duplexes targeting each ESCRT component and selected the siRNAs with the highest knockdown efficiency for subsequent experiments (data not shown). Next, we individually knocked down each ESCRT component or simultaneously knocked down multiple members in each ESCRT subcomplex and determined their effects on the cell viability of MARC-145 cells. As shown in Fig. 1A, the indicated siRNAs were nontoxic to the cells (cell viability, >90%). Subsequently, MARC-145 cells were transfected with the indicated siRNAs or siRNA-negative control (siNC) for 36 h and then inoculated with PRRSV at a multiplicity of infection (MOI) of 0.5. At 24 h postinfection (hpi), the cells were collected to evaluate PRRSV infection by detecting the intracellular viral RNA abundance with quantitative real-time PCR (RT-qPCR) and extracellular progeny titers via assessing 50% tissue culture infected dose (TCID_50_), respectively. In Fig. 1B and C, knockdown of the ESCRT-0 components exhibited no or moderate effects on PRRSV infection. In contrast, PRRSV infection was significantly suppressed with knockdown of the components from ESCRT-I (TSG101, VPS28, VPS37A, and MVB12B), the ESCRT-II (ELL-associated protein 20 [EAP20]), the ESCRT-III (changed MVB protein 2A/B [CHMP2A/B], CHMP3, CHMP4C, CHMP5, CHMP6, and CHMP7), and the ESCRT-associated protein (VPS 20 associated 1 [VTA1]). These results illustrate that certain components of the ESCRT system are important for PRRSV infection.

### TSG101 is required for PRRSV infection.

**FIG 2:**
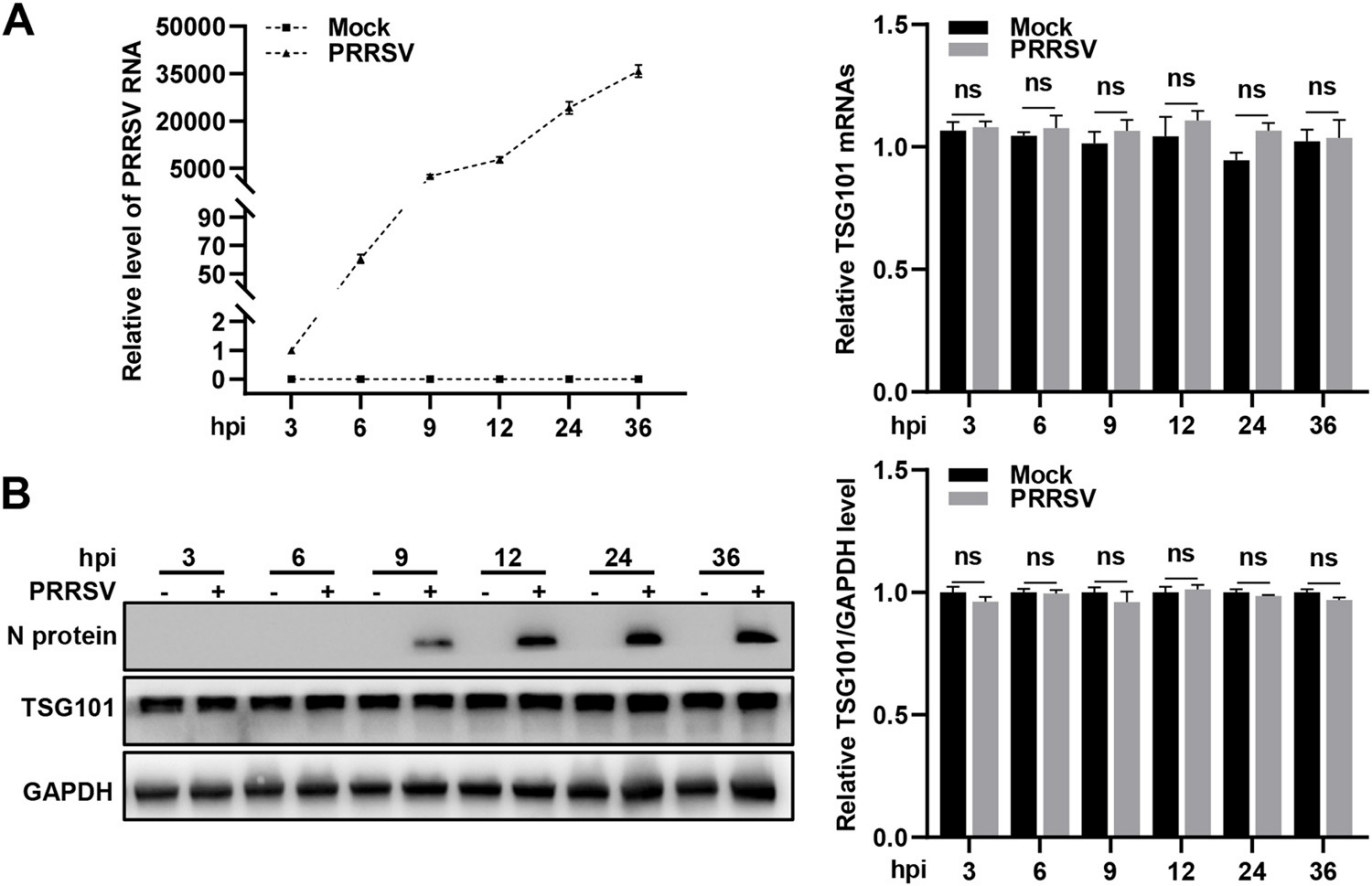
TSG101 expression is stable during PRRSV infection. (A-B) MARC-145 cells were infected with PRRSV (MOI = 1) or mock infected, and the samples were collected at the indicated time points (3, 6, 9, 12, 24, and 36 hpi). (A) The relative PRRSV RNA and TSG101 mRNA abundance in MARC-145 cells were analyzed using RT-qPCR. (B) Endogenous TSG101 protein expression was detected by IB. The grayscale of TSG101 was quantified using ImageJ software and normalized to GAPDH, where the value of the mock-infected cells in each group was normalized to 1.0. The data represent means ± the SEM from three independent experiments. Statistical analysis was carried out using the Student *t* test. ns, not significant (*P* > 0.05).

TSG101 was previously shown to be involved in PRRSV infection (42) and identified as the upstream protein among the ESCRT components important for PRRSV infection in the present study (Fig. 1). Therefore, it was chosen for further investigation in this work. To understand how TSG101 was coopted during PRRSV infection, we examined the transcription and protein levels of endogenous TSG101. MARC-145 cells were infected with PRRSV or mock infected and then assessed by RT-qPCR and immunoblotting (IB) at the indicated time points (3, 6, 9, 12, 24, and 36 hpi). Figure 2 shows that the transcription and protein levels of TSG101 were stable during PRRSV infection and showed no significant differences from the ones in the mock-infected cells.

**FIG 3:**
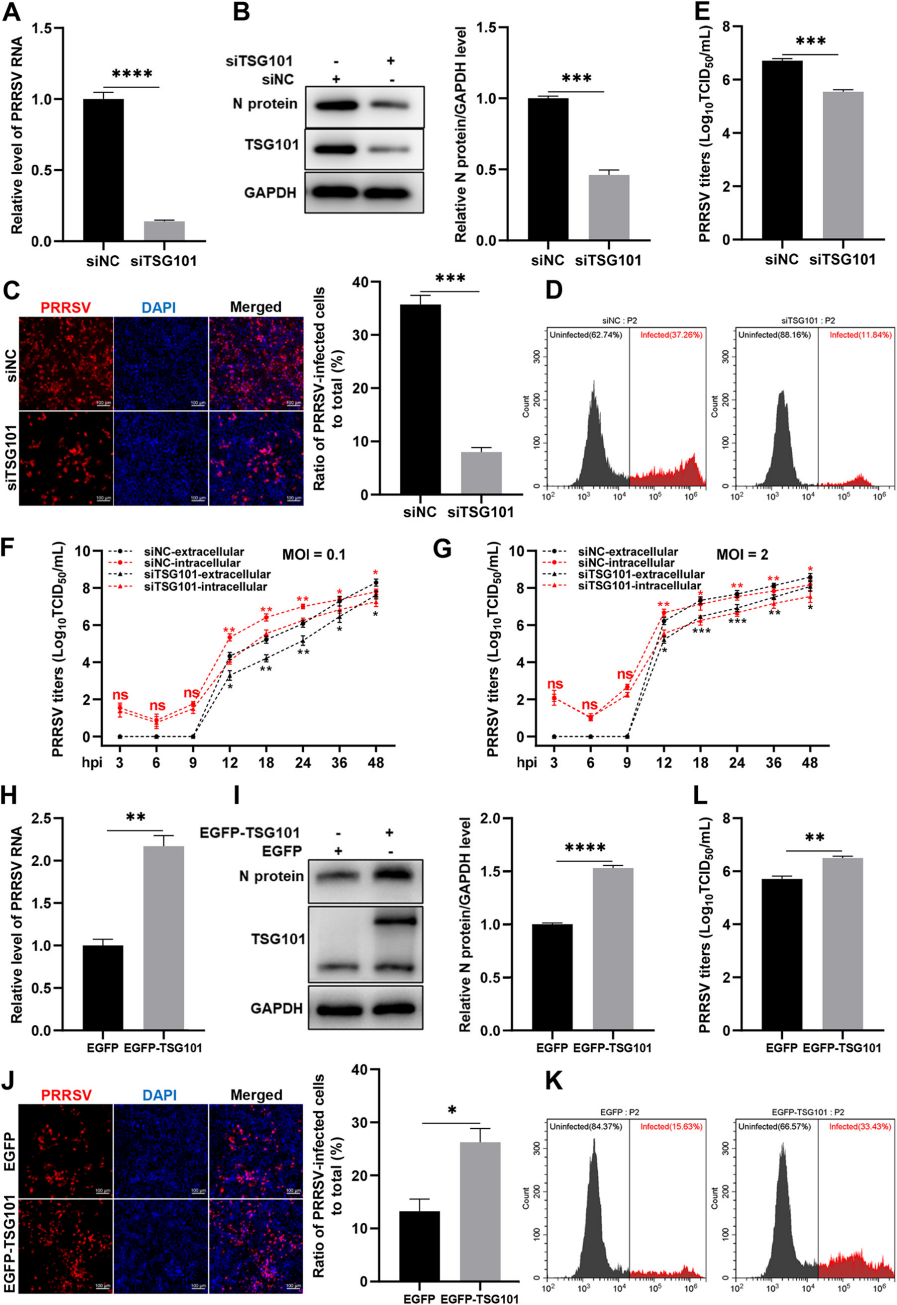
TSG101 is important for PRRSV infection. (A to E) Knockdown of TSG101 inhibited PRRSV infection. The MARC-145 cells seeded into 24-well plates were transfected with 50 nM siRNA targeting TSG101 or siNC for 36 h and then inoculated with PRRSV at an MOI of 0.5 for 24 h. The infected cells were harvested for detection of PRRSV RNA abundance using RT-qPCR (A), endogenous TSG101 or PRRSV N protein expression using IB (B), PRRSV infectivity using IFA (C) or FCM (D), and extracellular progeny viral titers by assessing TCID_50_ (E). (F and G) The *TSG101* knockdown cells or the control cells were infected with 0.1 MOI (F) or 2 MOI (G) PRRSV. After incubation at 37°C for 1 h, the cells were washed thoroughly with PBS to remove the uninternalized virions and then added with the fresh DMEM containing 2% FBS. The cells and supernatants were harvested at 3, 6, 9, 12, 18, 24, 36, and 48 hpi, respectively. Both the intracellular and extracellular virus titers at different time points were evaluated by assessing TCID_50_. (H to L) Overexpression of TSG101 increased PRRSV infection. MARC-145 cells were transfected with the vector expressing EGFP or EGFP-TSG101 for 36 h and then inoculated with PRRSV at an MOI of 0.1 for 24 h. The infected cells were collected for detection of PRRSV RNA abundance using RT-qPCR (H), TSG101 or PRRSV N protein expression using IB (I), PRRSV infectivity using IFA (J) or FCM (K), and the extracellular progeny virus titers by detecting TCID_50_ (L). The relative intensity of viral N protein was quantified by ImageJ software and normalized to GAPDH. IFA images were taken by confocal microscopy, and the numbers of PRRSV N-expressed (red) and DAPI-stained (blue) cells were counted using ImageJ software. The infection rate is expressed as PRRSV N-expressed cells/DAPI-stained cells. Scale bars, 100 μm. Data represent means ± the SEM from three independent experiments. Statistical analysis was carried out using the Student *t* test. *, *P* < 0.05; **, *P* < 0.01; ***, *P* < 0.001; ****, *P* < 0.0001; ns, not significant (*P* > 0.05).

To confirm the role of TSG101 in PRRSV infection, we first knocked down endogenous TSG101 in MARC-145 cells. *TSG101* knockdown significantly decreased the abundance of PRRSV RNA (∼85%, Fig. 3A), the expression level of viral N protein (∼55%, Fig. 3B), the viral infectivity (∼75% detected by indirect immunofluorescence assay [IFA] in Fig. 3C; ∼70% detected by flow cytometry [FCM] in Fig. 3D), and the extracellular progeny viral titers (∼1.2 log_10_TCID_50_/mL, corresponding to ∼16-fold, Fig. 3E) at 24 hpi. In order to explore the dynamics of TSG101-PRRSV interaction throughout different steps of the virus life cycle, we additionally determined the intracellular and extracellular viral titers at an MOI of 0.1 (Fig. 3F) and an MOI of 2 (Fig. 3G) in the control and *TSG101* knockdown cells at different time points, respectively. The TCID_50_ data showed that knockdown of TSG101 remarkably decreased both the intracellular and the extracellular viral titers of PRRSV after 9 hpi but had no effect before 9 hpi.

To further demonstrate its importance in PRRSV infection, we overexpressed exogenous TSG101 (enhanced green fluorescent protein [EGFP]-TSG101) in MARC-145 cells. TSG101 overexpression increased PRRSV RNA abundance (∼120%, Fig. 3H), N protein expression (∼60%, Fig. 3I), viral infectivity (∼110% detected by IFA in Fig. 3J; ∼115% detected by FCM in Fig. 3K), and extracellular progeny titers (∼1 log_10_TCID_50_/mL, corresponding to ∼10-fold, Fig. 3L) at 24 hpi. Together, these results provide evidence that TSG101 contributes to PRRSV infection in MARC-145 cells.

### TSG101 is not involved in viral attachment, internalization, RNA replication, and N protein translation during the PRRSV first life cycle.

**FIG 4:**
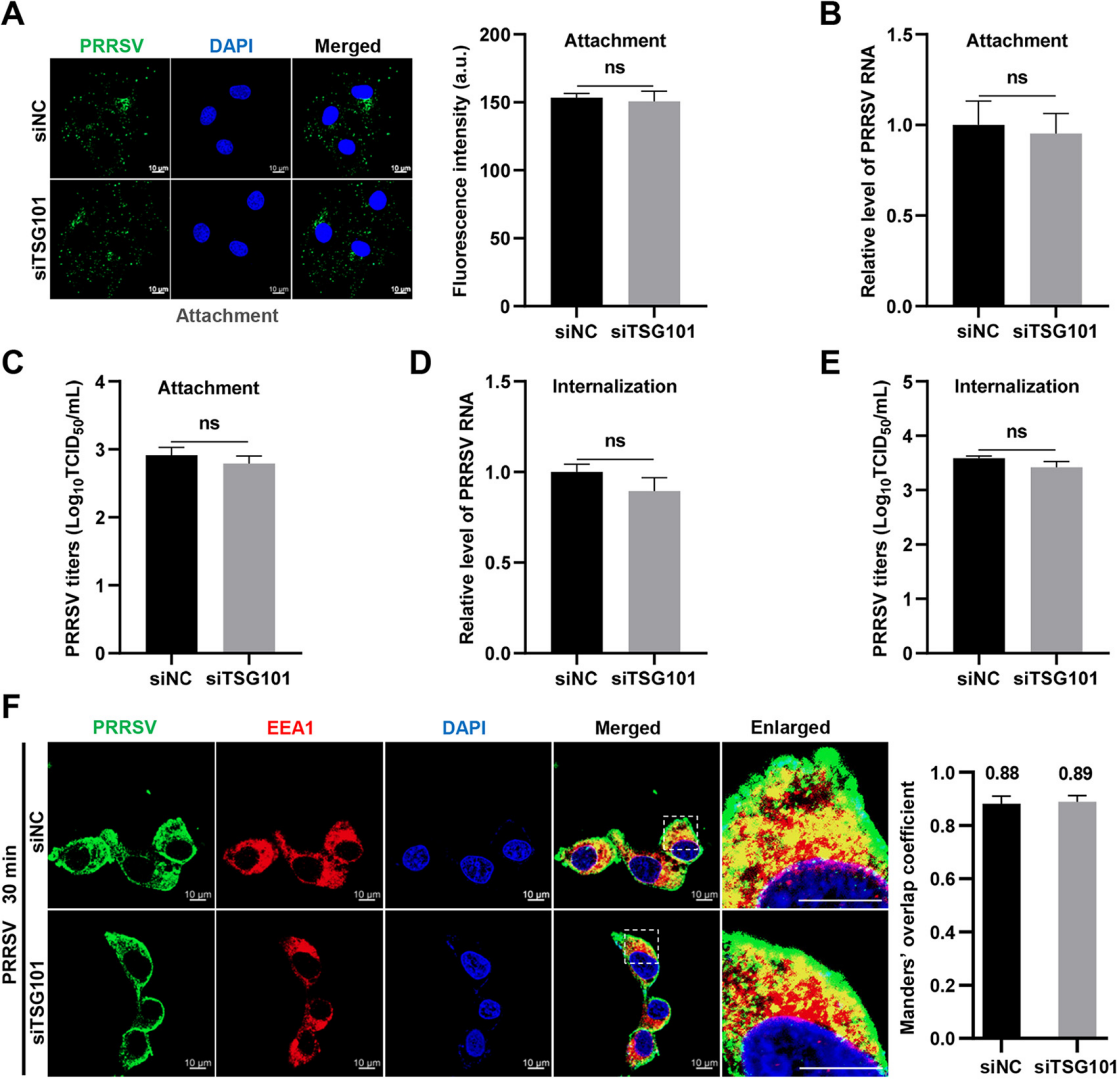
TSG101 is not involved in PRRSV attachment and internalization. (A to C) Knockdown of TSG101 did not affect PRRSV attachment. The siTSG101- or siNC-transfected MARC-145 cells were infected with PRRSV at an MOI of 10 and cultured at 4°C for 1 h. After adsorption, the unbound viruses were extensively washed away with ice-cold PBS. (A) The cell-bound PRRSV virions were assessed with mouse anti-GP5 MAb (green) by confocal microscopy. The total fluorescence intensity of GP5 was calculated using ImageJ software. Representative images are shown. Scale bars, 10 μm. (B) The cell-attached PRRSV virions were quantified by RT-qPCR. (C) After washes, the cells were subjected to three freeze-thaw cycles, and the cell-attached viral titers were assessed by detecting TCID_50_. (D to F) Knockdown of TSG101 did not affect PRRSV internalization. The siRNA-transfected MARC-145 cells were infected with PRRSV at an MOI of 10 and incubated at 4°C for 1 h. The unbound PRRSV virions were washed away with ice-cold PBS and the cells were switched to 37°C for 1 h. After washes, the internalized virions were quantified by RT-qPCR (D) and by assessing TCID_50_ (E). (F) The MARC-145 cells seeded in glass dishes were infected with PRRSV at 10 MOI and cultured at 4°C for 1 h and then shifted to 37°C for 30 min after washes. The cells were stained with mouse anti-PRRSV N protein MAb (green) and rabbit anti-EEA1 pAbs (red). Nuclei were stained with DAPI (blue). The colocalization of PRRSV N protein with EEA1 was observed using confocal microscopy. Images were taken at a 630× magnification and representative as a single slice of a stack from three independent experiments. Representative images are shown. Scale bars, 10 μm. The colocalization was assessed by determination of Manders’ overlap coefficient using the JaCoP plugin in ImageJ software. Manders’ overlap coefficient (>0.6) indicates an actual overlap of the signals and is considered to represent the true degree of colocalization. The mean value ± the SEM is representative of three individual enlarged pictures. Differences between groups were assessed by the Student *t* test. ns, not significant (*P* > 0.05).

To assess the involvement of TSG101 in PRRSV life cycle, we tested the effects of *TSG101* knockdown on different stages during viral infection. The siRNA-transfected MARC-145 cells were inoculated with PRRSV at an MOI of 10 and incubated at 4°C for 1 h. After adsorption, the unbound viruses were washed away, and the bound ones were visualized by confocal microscopy to analyze viral attachment. As shown in Fig. 4A, there was no significant difference in the amount of bound PRRSV virions between siTSG101- and siNC-transfected cells. Furthermore, the cell-bound PRRSV RNA abundance and titers were not decreased by *TSG101* knockdown (Fig. 4B and C), which is consistent with the fluorescence intensity data.

We subsequently determined whether TSG101 played a role in PRRSV internalization. PRRSV was inoculated in siTSG101- or siNC-transfected cells at 4°C for 1 h and at 37°C for another 1 h to allow internalization. The RT-qPCR and titration assays showed that TSG101 had no effect on PRRSV internalization (Fig. 4D and E). A previous report has shown that PRRSV virions were internalized into early endosomes for productive infection and colocalized with early endosomes (marked by early endosome antigen 1 [EEA1]) at 30 min postinfection (43). In Fig. 4F, *TSG101* knockdown was unable to block PRRSV internalization into early endosomes. The colocalization coefficient was expressed as Manders’ overlap coefficient, and the values were 0.88 and 0.89, respectively, indicating their colocalization (44).

**FIG 5:**
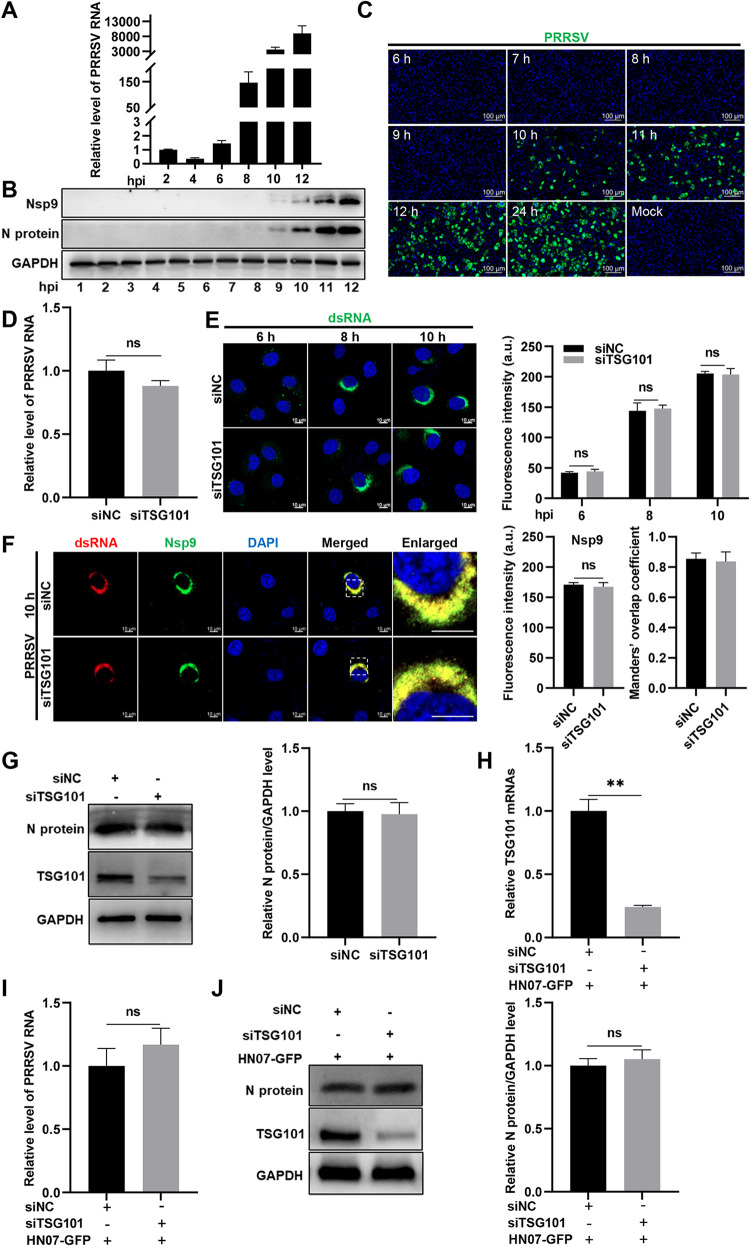
TSG101 is not involved in PRRSV RNA replication and N protein translation. (A to C) Determination of the first life cycle of PRRSV. The MARC-145 cells grown in 24-well plates were infected with PRRSV at an MOI of 2 at 37°C for 1 h. Then, the cells were washed thoroughly with PBS to remove uninternalized virions. The infected cells were cultured with 500 μL of DMEM containing 2% FBS for the indicated time periods. (A) The infected cells were collected every 2 h until at 12 hpi to assess the intracellular PRRSV RNA abundance using RT-qPCR. (B) The infected cells were collected every 1 h until at 12 hpi to monitor the newly translated PRRSV Nsp9 and N protein by IB. (C) The supernatant in each well was harvested every 1 h from at 6 hpi to at 12 hpi or at 24 hpi. The fresh MARC-145 cells grown in 24-well plates were infected with the collected supernatants for 36 h. IFA was performed to detect the fluorescence signal of virus stained with a mouse anti-PRRSV N protein MAb (green). Nuclei were stained with DAPI (blue). Representative images are shown. Scale bars, 100 μm. (D to G) TSG101 was not involved in PRRSV RNA replication and N protein translation in MARC-145 cells. The MARC-145 cells transfected with either siTSG101 or siNC were infected with PRRSV at an MOI of 1 for 10 h. The infected cells were harvested for detection of PRRSV RNA abundance using RT-qPCR (D), PRRSV dsRNA abundance (at 6, 8 and 10 hpi) using IFA (E), the Nsp9 fluorescence density and the colocalization between dsRNA and Nsp9 using confocal microscopy (F), and the TSG101 or PRRSV N protein expression using IB (G). The total fluorescence intensity of dsRNA was calculated using ImageJ software. The colocalization between dsRNA and Nsp9 was assessed by determination of Manders’ overlap coefficient using the JaCoP plugin in ImageJ software. The mean value ± the SEM is representative of three individual enlarged pictures. Representative images are shown. Scale bars, 10 μm. (H to J) TSG101 was not involved in RNA replication and N protein translation of PRRSV infectious clone. The HEK-293T cells grown in 12-well plates were transfected with siTSG101 or siNC for 24 h and then transfected with 2.5 μg of PRRSV infectious clone (HN07-GFP) for another 36 h. The transfected cells were harvested for detection of the abundance of TSG101 mRNA (H) or PRRSV RNA (I) using RT-qPCR, the expression of TSG101 or PRRSV N protein using IB (J). Data represent means ± the SEM from three independent experiments. Statistical analysis was carried out using the Student *t* test. **, *P* < 0.01; ns, not significant (*P* > 0.05).

Next, we delineated the kinetics of PRRSV viral RNA replication and protein expression during infection. As shown in Fig. 5A and B, PRRSV RNA abundance began to increase at 6 hpi, and viral RNA-dependent RNA-polymerase Nsp9 and N protein expression were initially detectable by IB at 9 hpi. The progeny virions in the supernatant were collected to assess the accurate time period of PRRSV first life cycle by IFA. In Fig. 5C, PRRSV progeny virions were not released into the supernatant until at 10 hpi. Considering the dynamics of PRRSV infection shown in Fig. 2 and 3, we consequently evaluated the involvement of TSG101 in PRRSV RNA replication and N protein translation at 10 hpi within the first life cycle. Knockdown of TSG101 did not alter the abundance of intracellular PRRSV RNA (Fig. 5D), the fluorescence intensity of double-stranded RNA (dsRNA; the intermediate of viral RNA synthesis) and Nsp9 (an essential component in the viral replication and transcription complex [RTC]; Fig. 5E and F), the colocalization between dsRNA and Nsp9 (Fig. 5F), and the level of newly translated N protein (Fig. 5G).

To further address the conclusion, a PRRSV nonsusceptible human embryonic kidney cell line (HEK-293T) was transfected with siTSG101 or siNC for 24 h, followed by transfection with an infectious clone (GFP-HN07). After additional 36 h, the cells were collected, and the results showed that *TSG101* knockdown took no effect on PRRSV RNA replication and N protein expression (Fig. 5H to J). Taken together, these results suggest that TSG101 is not involved in viral attachment, internalization, RNA replication, and N protein translation during the PRRSV first life cycle.

### TSG101 is involved in virion formation during PRRSV assembly.

**FIG 6:**
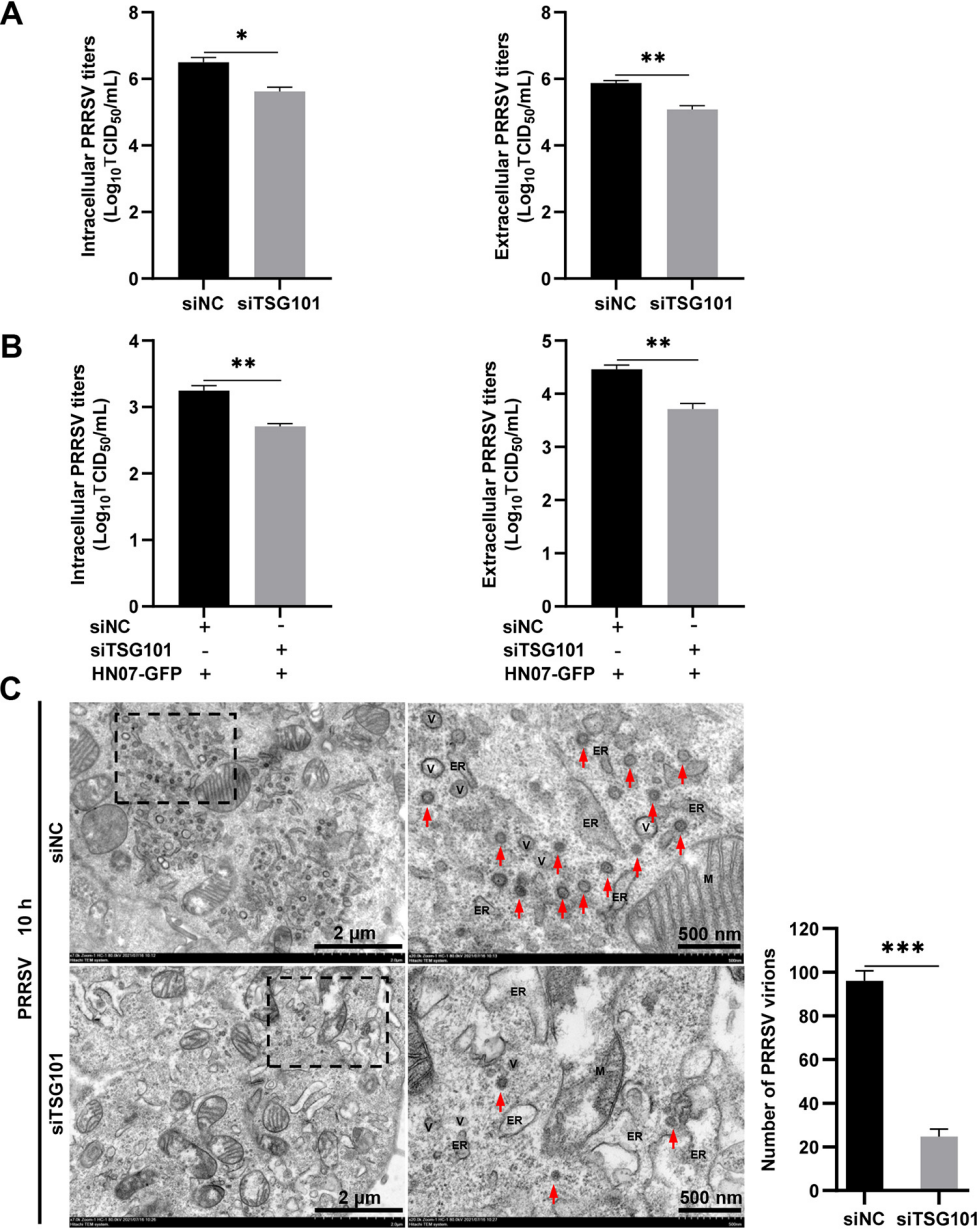
TSG101 is required for PRRSV virion formation. (A) MARC-145 cells transfected with siTSG101 or siNC were infected with PRRSV at an MOI of 1 for 10 h. The infected cells and supernatants were harvested separately for detection of the intracellular and extracellular progeny virus titers by assessing TCID_50_. (B) HEK-293T cells grown in 12-well plates were transfected with siTSG101 or siNC for 24 h and then transfected with 2.5 μg of PRRSV infectious clone (HN07-GFP) for another 36 h. The transfected cells and supernatants were harvested separately for detection of the intracellular and extracellular virus titers by detecting TCID_50_. (C) TEM analysis of PRRSV virion formation. The MARC-145 cells transfected with siTSG101 or siNC were infected with PRRSV at an MOI of 2 at 37°C for 10 h. The infected cells were fixed and investigated using 80-kV TEM according to standard procedures. Representative electron micrographs of the infected cells are shown. The image on the right panels showed an enlargement of the selected area within the rectangle. Low magnification: scale bars, 2 μm; high magnification: scale bars, 500 nm. M, mitochondrion; V, vesicle; ER, endoplasmic reticulum. Red arrows indicated the typical PRRSV virions. The number of virions per 50 μm^2^ areas was counted. Data represent means ± the SEM from three independent experiments. Statistical analysis was carried out using the Student *t* test. *, *P* < 0.05; **, *P* < 0.01; ***, *P* < 0.001; ns, not significant (*P* > 0.05).

Based on the results above, we hypothesized that TSG101 might be involved in PRRSV assembly and/or release. To test this hypothesis, we determined the effect of *TSG101* knockdown on PRRSV intracellular and extracellular viral titers at 10 hpi. As shown in Fig. 6A, both the intracellular and the extracellular viral titers were significantly decreased in siTSG101-transfected MARC-145 cells within the first life cycle. In addition, a significant reduction in the intracellular and extracellular viral titers was also observed in *TSG101* knockdown HEK-293T cells with PRRSV infectious clone transfection (Fig. 6B). These data suggested that TSG101 was involved in PRRSV virion formation during assembly at first.

To further verify whether *TSG101* knockdown influenced formation of PRRSV virions, transmission electron microscopy (TEM) was performed to observe PRRSV virion formation within the first life cycle in siTSG101- and siNC-transfected MARC-145 cells. Consistent with a previous report (45), a large number of dense and egg-shaped PRRSV virions with a diameter of 50 to 74 nm were monitored in the siNC-transfected cells. However, much fewer PRRSV virions were observed in the siTSG101-transfected ones (Fig. 6C). All of these results indicate that TSG101 plays an important role in PRRSV virion formation.

### TSG101 interacts with PRRSV N protein.

**FIG 7:**
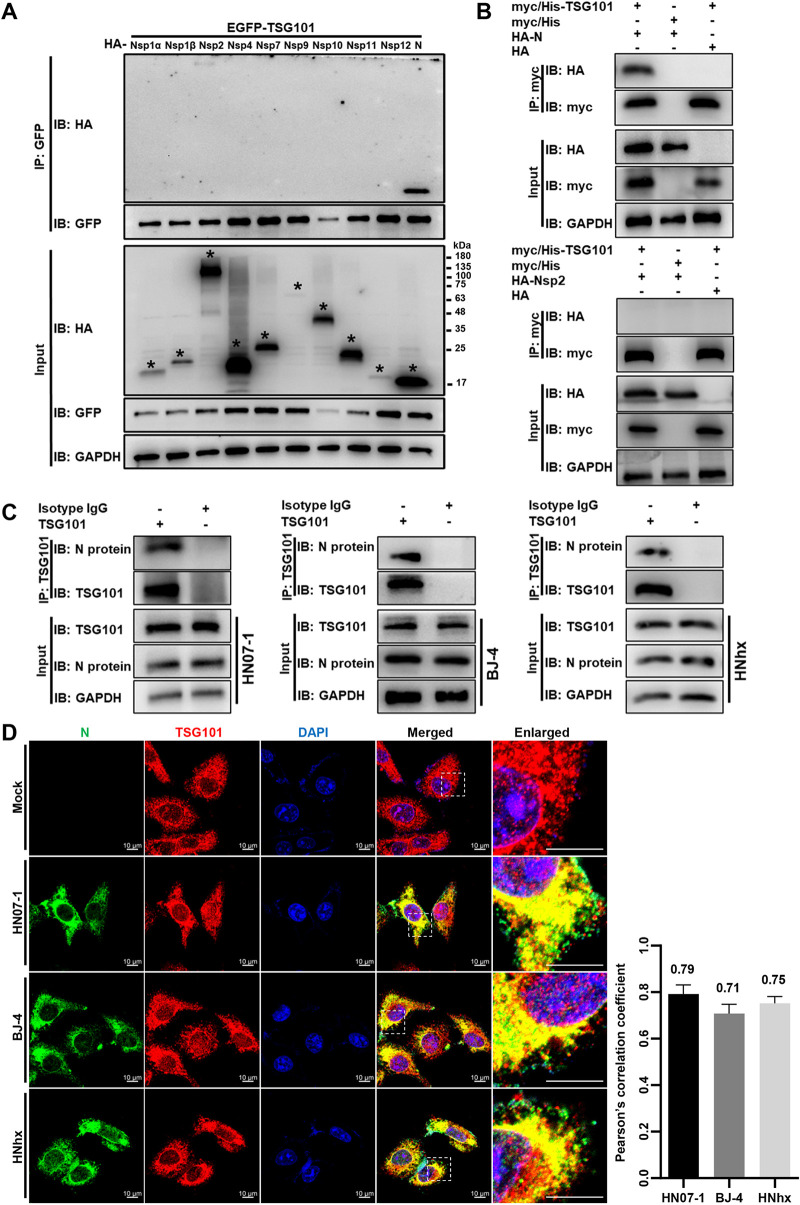
TSG101 interacts with PRRSV N protein. (A) Identification of PRRSV proteins interacting with TSG101 by GFP-pulldown assay. HEK-293T cells were coexpressed with EGFP-tagged TSG101 (EGFP-TSG101) and HA-tagged PRRSV proteins (HA-Nsp1α, -Nsp1β, -Nsp2, -Nsp4, -Nsp7, -Nsp9-12, or -N) at 37°C for 36 h. The cell lysates were immunoprecipitated with GFP-magarose beads and the TSG101 bound proteins were subjected to IB with a mouse anti-HA MAb. Asterisks marked the expressed HA-tagged PRRSV proteins. GAPDH was shown as an internal control. The images are representative of three independent experiments. (B) IP analysis of exogenous TSG101 binding to RRRSV N protein. The cell lysates were immunoprecipitated with Pierce anti-myc magnetic beads from the HEK-293T cells coexpressed with myc/His-TSG101 and HA-N protein, with myc/His-TSG101 and HA, or with myc/His and HA-N protein. The cells coexpressed with myc/His-TSG101 and HA-Nsp2 served as a negative control. Then the precipitated proteins were analyzed by IB with mouse anti-myc MAb or mouse anti-HA MAb. (C) IP analyses of endogenous TSG101 binding to N protein of different RRRSV strains in the infected cells. MARC-145 cells were infected with 10 MOI of RRRSV strains HN07-1 (left), BJ-4 (middle), and HNhx (right) at 37°C for 10 h and then lysed with NP-40 lysis buffer containing protease inhibitor cocktail. Using TSG101 as bait, the precipitated proteins in cell lysates were identified by IB with rabbit anti-PRRSV N protein pAbs. Isotype IgG antibody was used as a negative control. (D) Analyses of the colocalization between endogenous TSG101 (red) and N protein (green) of different RRRSV strains in the infected cells at 10 hpi. Nuclei were stained with DAPI (blue). Alexa Fluor 488-goat anti-rabbit IgG and Alexa Fluor 647-goat anti-mouse IgG were used as secondary antibodies. Images were acquired at a 630× magnification and representative as a single slice of a stack from three independent experiments. Representative images are shown. Scale bars, 10 μm. The colocalization was assessed with Pearson’s correlation coefficient using the JaCoP plugin in ImageJ software. Pearson’s correlation coefficient (>0.5) describes the correlation of the intensity distribution between channels. The mean value ± the SEM is representative of three individual enlarged pictures.

Next, we investigated the potential mechanisms by which TSG101 participated in PRRSV virion formation during assembly. As TSG101 has been reported to interact with Japanese encephalitis virus (JEV) protein to facilitate virion assembly (36), we screened which PRRSV protein(s) interacted with TSG101. HEK-293T cells were coexpressed with EGFP-tagged TSG101 and hemagglutinin (HA)-tagged PRRSV proteins and applied to GFP-pulldown assays. HA-tagged Nsp1α, Nsp1β, Nsp2, Nsp4, Nsp7, Nsp9-Nsp12, and N protein were expressed (Fig. 7A), and other viral proteins were not (data not shown). In Fig. 7A, TSG101 was shown to specially interact with PRRSV N protein. Immunoprecipitation (IP) further corroborated exogenous myc/His-TSG101 binding to HA-N protein (Fig. 7B, top panel) rather than HA-Nsp2 as a negative control (Fig. 7B, bottom panel). Moreover, the interaction between endogenous TSG101 and PRRSV N protein was also detected in the PRRSV-infected MARC-145 cells by IP (HN07-1; Fig. 7C, left panel) and confocal microscopy (HN07-1; Fig. 7D, second panel), respectively. The endogenous colocalization was quantified and expressed as Pearson’s correlation coefficient, and the mean value was 0.79, suggesting that there existed an interaction (44). Considering N protein variants among different PRRSV strains, we determined the interaction between endogenous TSG101 and the viral N protein from another two strains (BJ-4 and HNhx). The IP and confocal microscopy results demonstrated their interactions as well (Fig. 7C and D). These data demonstrate that TSG101 specifically interacts with PRRSV N protein.

### TSG101 is not essential for PRRSV nucleocapsid oligomerization.

**FIG 8:**
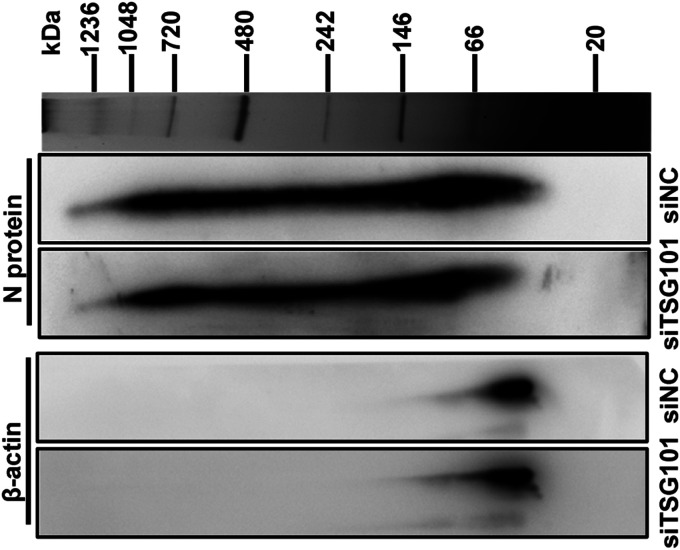
TSG101 is not essential for PRRSV nucleocapsid oligomerization. The MARC-145 cells transfected with siTSG101 or siNC were inoculated with PRRSV at an MOI of 5 at 37°C for 10 h. The infected cells were lysed and the nucleocapsid complexes were separated by a nondenatured NativePAGE 3%–12% Bis-Tris gels in the first dimension. After electrophoresis, the individual lanes were cut out of the NativePAGE gel and then the nucleocapsid complexes were separated by a two-dimensional denatured SDS-PAGE gel (NuPAGE 12% Bis-Tris gels with 2D-well). The nucleocapsid was detected by IB with rabbit anti-PRRSV N pAbs. β-actin was used as a loading control.

Earlier studies have confirmed that PRRSV N protein form into nucleocapsid via polymerization of monomeric and dimeric forms, namely, nucleocapsid oligomerization, which is critical for PRRSV virion formation (46, 47). Since TSG101 was shown to be involved in PRRSV virion formation and interact with the N protein, we detected whether *TSG101* knockdown affected the polymerization status of PRRSV N protein. Two-dimensional electrophoresis was conducted with native polyacrylamide gel electrophoresis (PAGE) in the first dimension and denatured, reduced sodium dodecyl sulfate-PAGE (SDS-PAGE) in the second one, as previously described (48). As shown in Fig. 8, the IB bands revealed a variety of monomeric to high-order oligomeric complexes (from >20 to 1,236 kDa), representing the intermediate and fully assembled nucleocapsids. A similar distribution pattern of nucleocapsid complexes was obviously observed in both siTSG101- and siNC-transfected cells, suggesting that TSG101 took no effect on the oligomerization of PRRSV N protein.

### *TSG101* knockdown affects the subcellular localization of PRRSV N protein.

**FIG 9:**
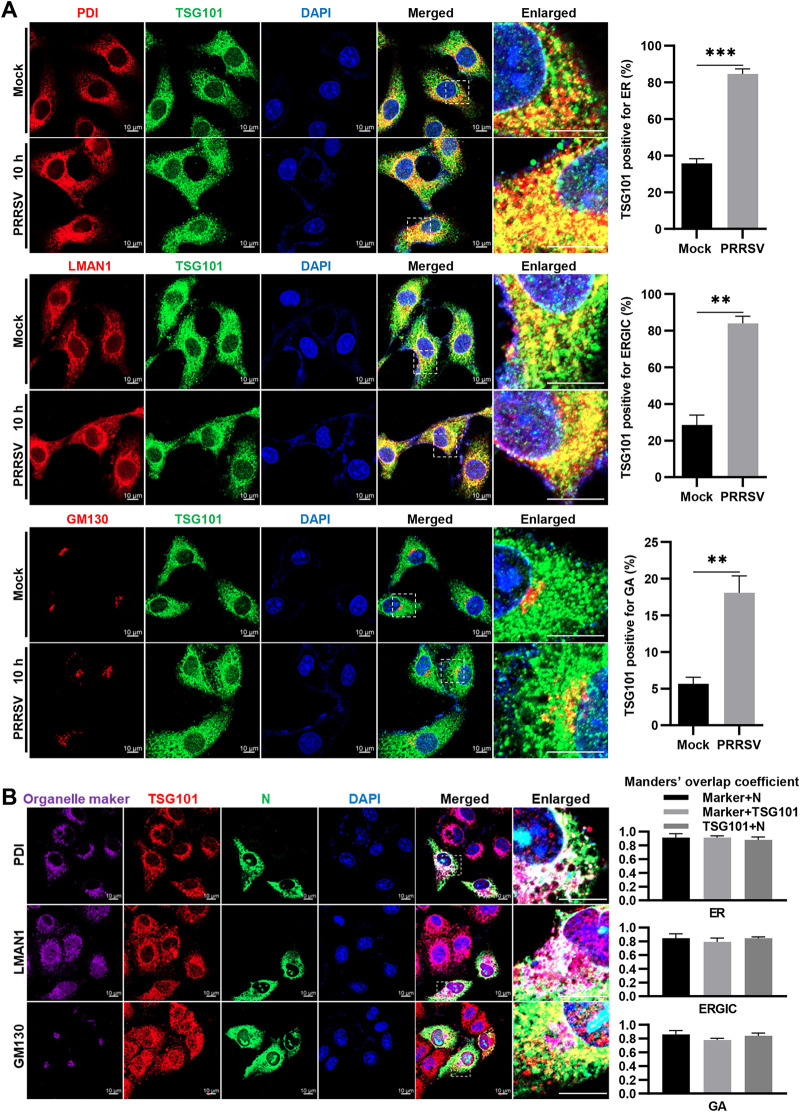
Subcellular localization of TSG101 and PRRSV N protein, along with the early secretory pathway. (A) Subcellular localization of TSG101 with the early secretory pathway. The MARC-145 cells seeded in glass dishes were infected with or without PRRSV (MOI = 10) at 37°C for 10 h. The cells were costained with mouse anti-TSG101 MAb (green) and rabbit anti-PDI MAb (red), rabbit anti-LMAN1 pAbs (red), or rabbit anti-GM130 MAb (red), respectively. Nuclei were stained with DAPI (blue). Alexa Fluor 647-goat anti-rabbit IgG and Alexa Fluor 488-goat anti-mouse IgG were used as secondary antibodies. Images were taken at a 630× magnification and representative as a single slice of a stack from three independent experiments. Representative images are shown. Scale bars, 10 μm. The colocalization was assessed by determination of Manders’ colocalization coefficient using the JaCoP plugin in ImageJ software. The coefficient represents the proportion of the colocalized fluorescence intensity of A protein with B protein to total fluorescence intensity of A protein. The mean value ± the SEM is representative of three individual enlarged pictures. Statistical analysis was carried out using the Student *t* test. **, *P* < 0.01; ***, *P* < 0.001. (B) Interaction between TSG101 and PRRSV N protein, along with the early secretory pathway. The MARC-145 cells seeded in glass dishes were infected with PRRSV (MOI = 10). After incubation at 37°C for 10 h, the cells were first costained with mouse anti-PRRSV N MAb (green) and rabbit anti-PDI MAb (purple), rabbit anti-LMAN1 pAbs (purple), or rabbit anti-GM130 MAb (purple), respectively. Subsequently, the cells were costained with Alexa Fluor 647-conjugated TSG101 MAb (red), Alexa Fluor 555-goat anti-rabbit IgG, and Alexa Fluor 488-goat anti-mouse IgG antibodies. Nuclei were stained with DAPI (blue). Images were taken at a 630× magnification and representative as a single slice of a stack from three independent experiments. Representative images are shown. Scale bars, 10 μm. The colocalization was assessed by determination of Manders’ overlap coefficient using the JaCoP plugin in ImageJ software. The mean value ± the SEM is representative of three individual enlarged pictures.

Correct subcellular localization of viral structural proteins in the early secretory pathway is another determinant for virion formation (49, 50). Therefore, we examined whether TSG101 took effect on the subcellular localization of PRRSV N protein with the early secretory pathway. We first carried out confocal microscopy to examine the localization of TSG101 with the early secretory pathway, including ER, the ER-Golgi intermediate compartment (ERGIC), and GA in PRRSV-infected MARC-145 cells at 10 hpi. In parallel, the mock-infected cells were monitored as control. As shown in Fig. 9A, TSG101 colocalized with protein disulfide isomerase (PDI)-labeled ER, lectin mannose binding 1 (LMAN1)-labeled ERGIC, and Golgi matrix protein 130 (GM130)-labeled GA in both mock- and PRRSV-infected cells. Interestingly, noticeable increases were observed in the percentage of TSG101-positive staining for ER (from ∼35% to ∼85%), ERGIC (from ∼30% to ∼85%), and GA (from ∼5% to ∼20%) in the PRRSV-infected cells compared to that in the mock-infected ones. In the meantime, we performed a triple colocalization analysis of TSG101, PRRSV N protein, and these three organelle makers by confocal microscopy. The fluorescent images showed that TSG101 interacted with PRRSV N protein, along with ER, ERGIC, and GA (Fig. 9B).

**FIG 10:**
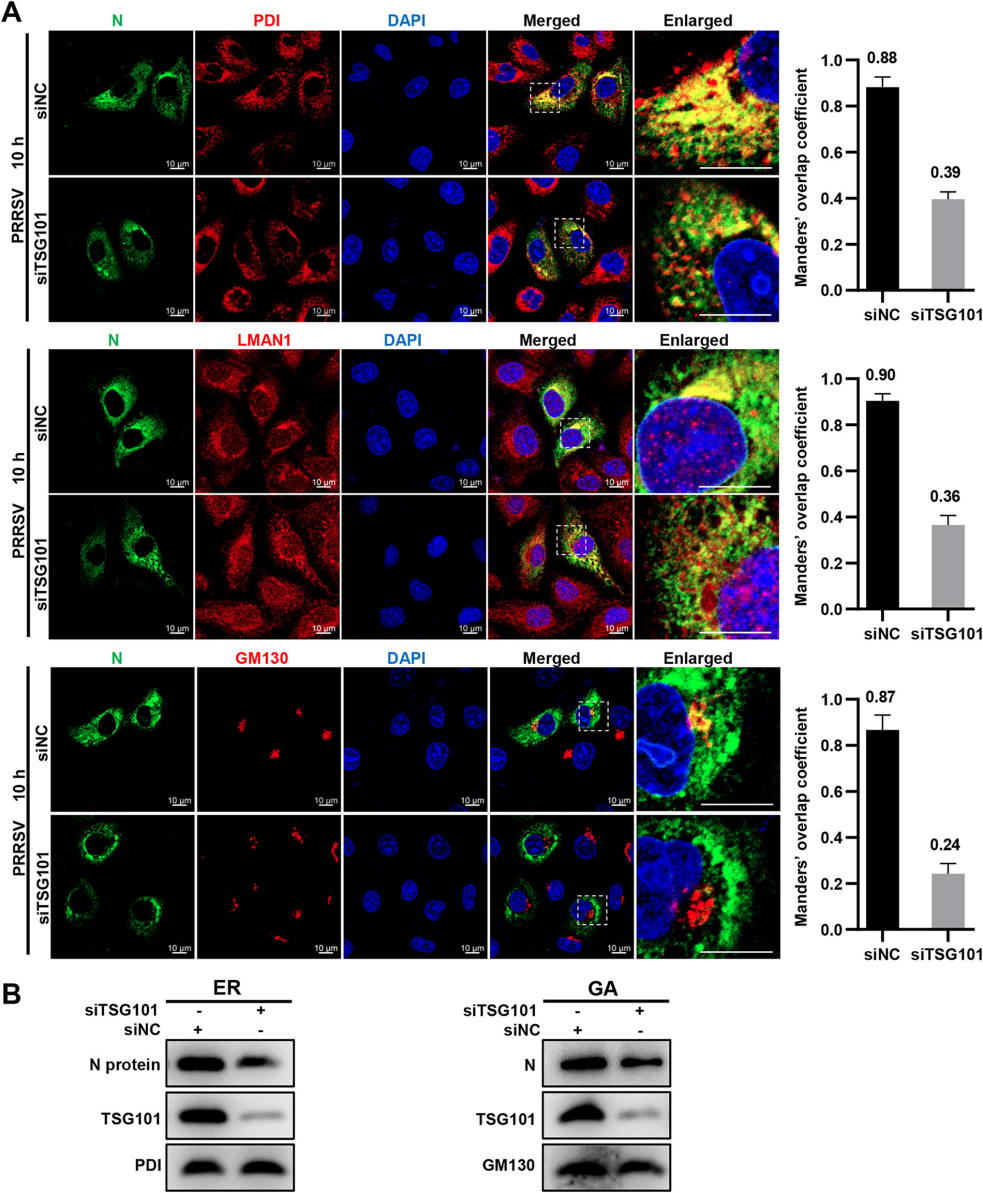
*TSG101* knockdown affects the subcellular localization of PRRSV N protein with the early secretory pathway. (A) *TSG101* knockdown impaired the subcellular distribution of PRRSV N protein. The MARC-145 cells seeded in glass dishes were transfected with either siTSG101 or siNC for 36 h and then infected with PRRSV (MOI = 10) at 37°C for 10 h. The cells were costained with mouse anti-PRRSV N MAb (green), rabbit anti-PDI MAb (red), rabbit anti-LMAN1 pAbs (red), or rabbit anti-GM130 MAb (red). Nuclei were stained with DAPI (blue). Alexa Fluor 647-goat anti-rabbit IgG and Alexa Fluor 488-goat anti-mouse IgG were used as secondary antibodies. Images were taken at a 630× magnification and representative as a single slice of a stack from three independent experiments. Representative images are shown. Scale bars, 10 μm. The colocalization was assessed by determination of Manders’ overlap coefficient using the JaCoP plugin in ImageJ software. The mean value ± the SEM is representative of three individual enlarged pictures. (B) IB analyses of ER/GA-enriched proteins. The MARC-145 cells seeded in 100-mm dishes were transfected with either siTSG101 or siNC for 36 h and infected with PRRSV (MOI = 10) at 37°C for 10 h. The cells were then collected and subjected to ER/GA protein extraction assay using a Minute ER/GA enrichment kit, respectively. The extracted proteins were subsequently examined by IB and stained with rabbit anti-TSG101 pAbs or rabbit anti-PRRSV N pAbs, along with rabbit anti-PDI MAb or rabbit anti-GM130 MAb as a loading control.

We subsequently measured the effect of *TSG101* knockdown on the subcellular localization of PRRSV N protein. As shown in Fig. 10A, the colocalization signal of PRRSV N protein with ER marker PDI, the ERGIC marker LMAN1, and GA marker GM130 were all decreased by *TSG101* knockdown. Manders’ overlap coefficient analysis also showed the decreased colocalization between N protein and ER (the mean value decreased from 0.88 to 0.39), ERGIC (from 0.90 to 0.36), and GA (from 0.87 to 0.24). To substantiate these observations, total proteins of ER and GA were enriched using commercial extraction kits to detect the abundance of PRRSV N protein at 10 hpi, respectively. As shown in Fig. 10B, PRRSV N protein abundance was significantly lowered in both the ER and GA extracts upon *TSG101* knockdown, in agreement with the confocal microscopy results. Therefore, we conclude that knockdown of TSG101 affects the subcellular localization of PRRSV N protein along with the early secretory pathway.

### TSG101 is important for PRRSV infection in CRL-2843-CD163 cells.

**FIG 11:**
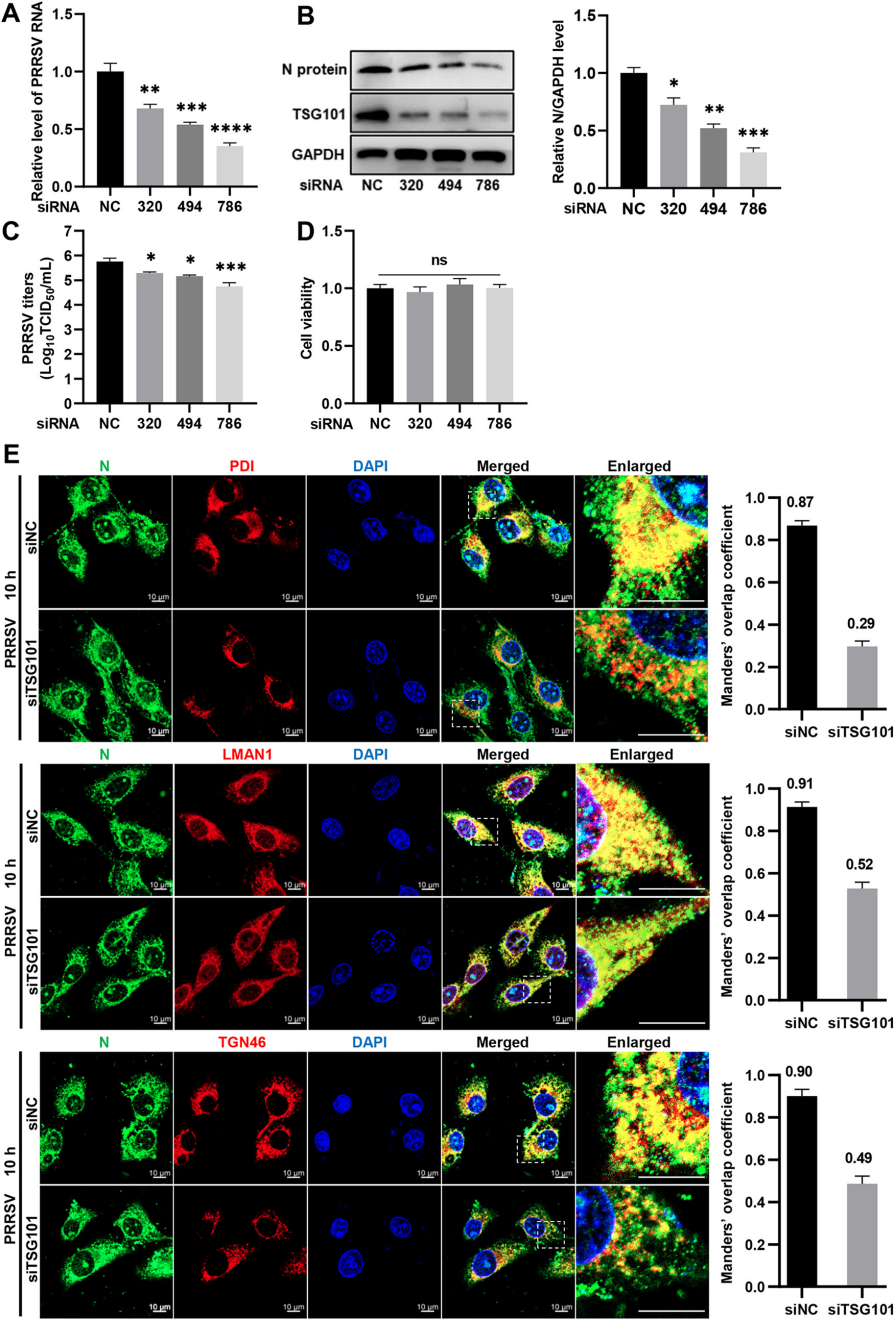
TSG101 is important for PRRSV infection in CRL-2843-CD163 cells. (A to C) Knockdown of TSG101 inhibited PRRSV infection. CRL-2843-CD163 cells were transfected with 50 nM siRNAs against pig TSG101 (siTSG101-320, siTSG101-494, and siTSG101-786) or siNC for 36 h and then inoculated with PRRSV (MOI = 0.5) at 37°C for additional 24 h. The infected cells were harvested for detection of PRRSV RNA abundance using RT-qPCR (A), endogenous TSG101 or PRRSV N protein expression using IB (B), and the extracellular progeny virus titers by detecting TCID_50_ (C). (D) Cytotoxicities of the indicated siRNAs. The CRL-2843-CD163 cells grown in 96-well plates were transfected with the indicated siRNAs (50 nM) for 60 h, and the cell viabilities were evaluated by performing a CellTiter 96 AQueous One solution cell proliferation assay. The cell viability in siNC-transfected cells was set to 1.0. Data represent means ± the SEM from three independent experiments. Statistical analysis was carried out using the Student *t* test. *, *P* < 0.05; **, *P* < 0.01; ***, *P* < 0.001; ****, *P* < 0.0001; ns, not significant (*P* > 0.05). (E) Confocal microscopy analyses of the colocalization of PRRSV N protein with subcellular markers (PDI for ER, LMAN1 for ERGIC, and TGN46 for GA). The CRL-2843-CD163 cells seeded in glass dishes were transfected with either siTSG101-786 or siNC for 36 h and then infected with PRRSV (MOI = 20) at 37°C for 10 h. The cells were stained with mouse anti-PRRSV N MAb (green), rabbit anti-PDI MAb (red), rabbit anti-LMAN1 pAbs (red), or rabbit anti-TGN46 pAbs (red). Nuclei were stained with DAPI (blue). Alexa Fluor 647-goat anti-rabbit IgG and Alexa Fluor 488-goat anti-mouse IgG were used as secondary antibodies. Images were taken at a 630× magnification and are representative as a single slice of a stack from three independent experiments. Representative images are shown. Scale bars, 10 μm. The colocalization was assessed by determination of Manders’ overlap coefficient using the JaCoP plugin in ImageJ software. The mean value ± the SEM is representative of three individual enlarged pictures.

As PAMs are primary *in vivo* target cells for PRRSV infection, we checked whether TSG101 played the same role during the viral infection in CRL-2843-CD163 cells, which are immortalized PAMs stably expressing PRRSV indispensable receptor CD163 (51, 52). Our results showed that knockdown of TSG101 significantly decreased PRRSV RNA abundance (Fig. 11A), N protein expression (Fig. 11B), and progeny viral titers (Fig. 11C) at 24 hpi, where all the three transfected-siRNAs were nontoxic to the cells (Fig. 11D). Importantly, *TSG101* knockdown affected the colocalization of PRRSV N protein with early secretory pathway (PDI for ER, LMAN1 for ERGIC, and trans-Golgi network protein 46 [TGN46] for GA) in the PRRSV first life cycle (Fig. 11E). A significant reduction in their colocalizations was indicated by Manders’ overlap coefficient (the mean value decreased from 0.87 to 0.29 for N-ER, from 0.91 to 0.52 for N-ERGIC, and from 0.90 to 0.49 for N-GA). Collectively, these results show that TSG101 takes effect on PRRSV infection both in MARC-145 and CRL-2843-CD163 cells.

## DISCUSSION

PRRSV exploits host cellular machinery for its proliferation, and therefore host factors required for PRRSV infection may be targeted for the development of antiviral agents. Despite tremendous progress in elucidation of PRRSV life cycle (53–55), an in-depth understanding of PRRSV assembly is still missing. In the present study, we unraveled the importance of TSG101, an ESCRT-I component, in PRRSV assembly for the first time.

Here, we first performed a siRNA-based screening of 30 ESCRT proteins and identified that PRRSV only utilized a part of ESCRT components for its infection, which are even in the same subcomplex (Fig. 1). Dengue virus and JEV were also found to exploit only TSG101, CHMP2/3, and CHMP4 family members in the ESCRT system for infection through systematic siRNA screening (40). In addition, equine infectious anemia virus usurps ALG-2 interacting protein X (ALIX), CHMP2A/B, CHMP4A/B, and VPS4 to release from host cells (56), and the release of murine leukemia virus only requires TSG101, CHMP2A, and CHMP4B (57). The reason for the specific usage of the ESCRT system needs to be clarified in future.

Among the ESCRT proteins important for PRRSV infection, TSG101 was identified as the most upstream factor. A previous study has also confirmed its role in PRRSV infection (42). Therefore, TSG101 was selected for further study in this work. With knockdown and overexpression of TSG101, we further confirmed its importance in PRRSV infection (Fig. 3). Viruses usually regulate the expression levels of host cellular proteins for their own benefits. Intriguingly, the transcription and protein levels of TSG101 kept stable in both PRRSV- and mock-infected cells (Fig. 2). Similar to our results, JEV proliferation does not change the cellular TSG101 expression to promote viral infection (36).

TSG101 is homologous to the E2 ubiquitin ligase that regulates ubiquitinated protein levels via its ubiquitin-binding activity. This activity has been reported to be involved in human papillomavirus and human immunodeficiency virus 1 infection (37, 58, 59). We measured whether the activity was required for PRRSV infection using a specific inhibitor tenatoprazole (40). Interestingly, our study showed that TSG101 facilitated the viral infection independent of ubiquitin-binding activity (data not shown).

We then attempted to characterize which stage of PRRSV life cycle is targeted by TSG101. We first addressed that *TSG101* knockdown did not influence attachment or internalization of PRRSV (Fig. 4). We next evaluated its role in the PRRSV first life cycle and found that *TSG101* knockdown resulted in a significant decrease in PRRSV intracellular virion formation rather than viral RNA replication and N protein expression (Fig. 5 and 6).

Virus assembly is a complex process and coordinates interactions between viral proteins and host cellular factors. The fundamental function of PRRSV N protein is to form the viral capsid through covalent and noncovalent interactions, and encapsulate the viral genome (46, 47). In addition, it interacts with a variety of host cellular factors, such as fibrillarin (60), PARP-1 (61), DHX9 (62), MOV10 (63), S100A9 (64), and TRIM25 (65), to modulate the viral infection. In Fig. 7, we carried out different assays to identify that TSG101 interacted with N protein from three different PRRSV strains prevalent in China. Previous literatures have reported that TSG101 directly binds to viral proteins containing the classical late-domain motifs (P[T/S]AP) (29, 37–39). Intriguingly, amino acid sequence analysis showed that there were no classical late-domain motifs in PRRSV N protein. In fact, increasing evidences show that TSG101 binds to viral proteins independent of late-domain motifs or dependent of novel nonclassical motifs (35, 36, 66). We will explore the interaction details between TSG101 and PRRSV N protein.

We next attempted to decipher the mechanism by which TSG101 contributed to PRRSV virion formation during assembly. The oligomerized N proteins support the essential and fundamental components for PRRSV virions (46). However, we found that knockdown of TSG101 did not affect the oligomerization of N protein (Fig. 8). Alternatively, the nucleocapsid must be translocated to appropriate compartment and packaged into enveloped virions. Therefore, its correct subcellular localization in the early secretory pathway, well established as a virus assembly site, is crucial for virion formation (49, 50). We found that more TSG101 was enriched in the early secretory pathway during PRRSV infection (Fig. 9A). Simultaneously, the interaction between TSG101 and PRRSV N protein was associated with this pathway (Fig. 9B). However, knockdown of TSG101 significantly decreased the subcellular distribution of N protein, along with the early secretory pathway both in MARC-145 (Fig. 10) and in CRL-2843-CD163 cells (Fig. 11). These results suggest a potential role for TSG101 in N protein intracellular transport during assembly. Previous studies have shown that TSG101 plays a role in transport of viral proteins to assembly sites. For example, TSG101 assists transport of the nucleocapsid to the assembly site of Marburg virus (39). In addition, TSG101 is identified as a component essential for the trafficking of influenza A virus HA to the plasma membrane prior to release (67). As a consequence, it is not surprising that TSG101 was involved in the trafficking of nucleocapsid to the assembly site of PRRSV. We speculated that knockdown of TSG101 led to the inaccurate transportation of nucleocapsid to assembly site, resulting in defective assembly of intracellular virions. Unfortunately, we have not determined where PRRSV completes assembly yet, which requires further exploration. In addition, we assumed that TSG101 might act as an adaptor to cooperate with other ESCRT components and induce membrane deformation to produce enveloped infectious virions (23).

Indeed, there are some issues left unsolved in the current work. The first one is the mechanisms of other identified ESCRT components involved in PRRSV infection. Second, since some PRRSV Nsps and structural proteins were not expressed in GFP-pulldown assays, we cannot exclude their interactions with TSG101. Third, how TSG101 was enriched in the early secretory pathway during PRRSV infection needs to be determined. Finally, the role of TSG101 in PRRSV release needs to be examined. All of these issues are being addressed in our laboratory.

**FIG 12:**
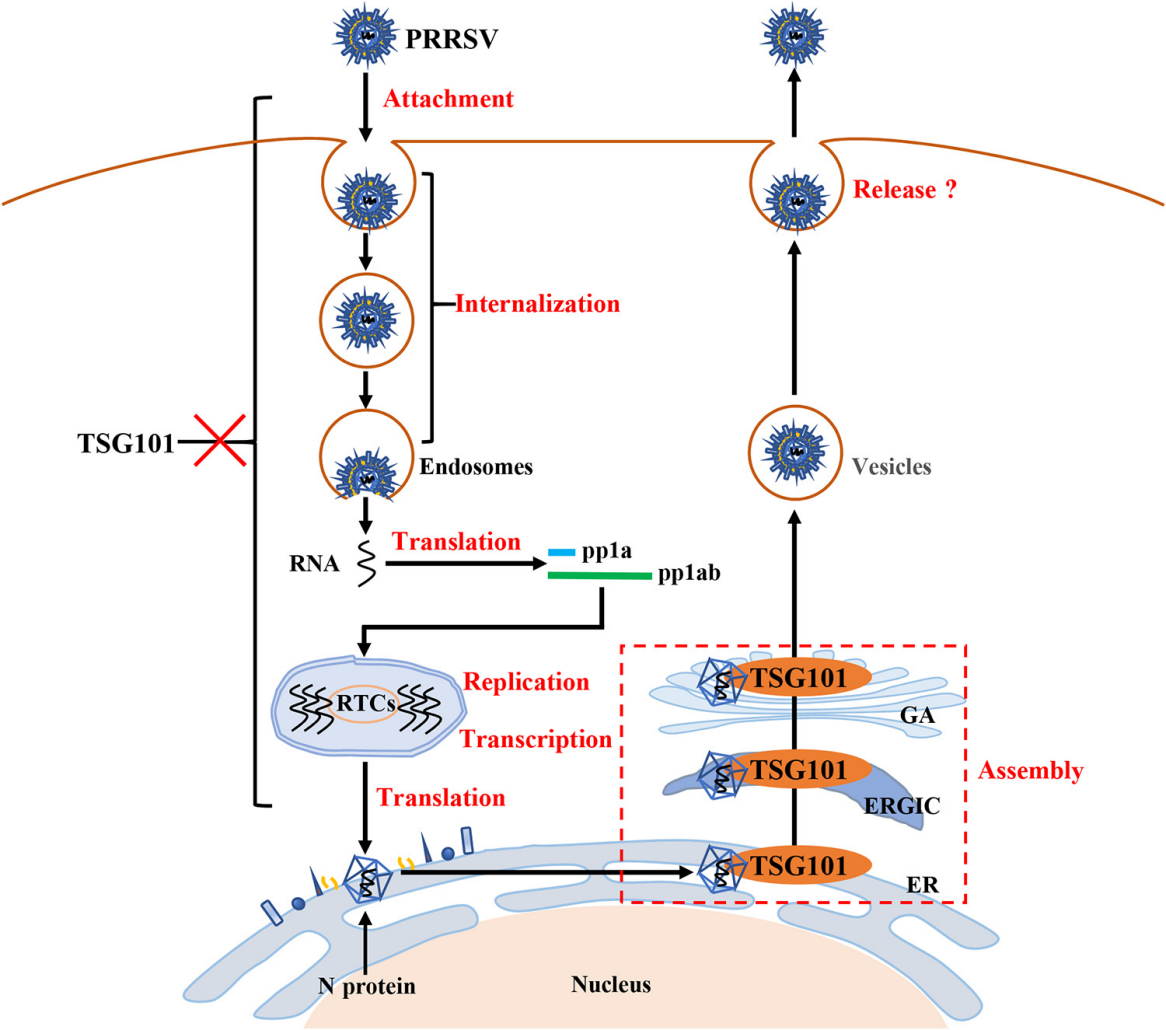
Schematic model depicting the role of TSG101 in PRRSV life cycle. TSG101 contributes to PRRSV virion formation during assembly rather than attachment, internalization, RNA replication, and N protein translation. Mechanistically, TSG101 interacts with PRRSV N protein and determines its subcellular location in the early secretory pathway.

Based on the results stated above, we propose a model to depict the involvement of TSG101 in PRRSV infection (Fig. 12). TSG101, as a novel host factor interacting with PRRSV N protein, is beneficial to PRRSV virion formation rather than attachment, internalization, RNA replication, and N protein translation during infection.

In summary, our work demonstrates that TSG101 contributes to PRRSV virion formation via interaction with the viral N protein along with the early secretory pathway. The results actually expand the knowledge of PRRSV infection and provide a novel therapeutic target for prevention and control of the virus. More importantly, considering its importance in multiple viral infections, TSG101 is promising to be developed as a broad-spectrum antiviral target.

## MATERIALS AND METHODS

### Cells and viruses.

MARC-145, CRL-2843-CD163, and HEK-293T cell lines were kept in our laboratory (68). MARC-145 and HEK-293T cells were cultured in Dulbecco modified Eagle medium (DMEM; catalog no. 12100; Solarbio, Beijing, China) supplemented with 10% heat-inactivated fetal bovine serum (FBS; catalog no. 10270-106; Gibco, Waltham, MA) and antibiotics (100 U/mL penicillin, 100 μg/mL streptomycin; catalog no. P1400; Solarbio) in a humidified 37°C, 5% CO_2_ incubator. CRL-2843-CD163 cells were routinely maintained in Roswell Park Memorial Institute 1640 medium (RPMI 1640; catalog no. 31800; Solarbio), supplemented with 10% FBS and antibiotics at 37°C in a 5% CO_2_ incubator.

PRRSV strain HN07-1 (GenBank accession no. KX766378.1) and NADC30-like strain HNhx (GenBank KX766379) were previously isolated by our laboratory (69, 70). PRRSV strain BJ-4 (GenBank AF331831) was kindly provided by Hanchun Yang from China Agricultural University. The infectious clone of HN07-1 (HN07-GFP) was constructed by inserting EGFP between PRRSV open reading frame 1b (ORF1b) and ORF2a through the reverse-genetics technique. The PRRSV strain used in the experiments was HN07-1 unless otherwise stated.

### Antibodies.

Rabbit anti-EEA1 polyclonal antibodies (pAbs; catalog no. 2411), rabbit anti-β-actin monoclonal antibody (MAb; catalog no. 2128), rabbit anti-PDI MAb (catalog no. 3501), rabbit anti-GM130 MAb (catalog no. 12480), and rabbit isotype IgG (catalog no. 2729) were all purchased from Cell Signaling Technology (Danvers, MA). Rabbit anti-TSG101 pAbs (catalog no. 28283-1-AP) and mouse anti-glyceraldehyde-3-phosphate dehydrogenase (GAPDH) MAb (catalog no. 60004-1-Ig) were purchased from Proteintech (Wuhan, China). Mouse anti-myc MAb (catalog no. M2002), mouse anti-HA MAb (catalog no. M2003), and mouse anti-GFP MAb (catalog no. M2004) were purchased from Ab-mart (Shanghai, China). Rabbit anti-LMAN1 pAbs (catalog no. bs-18304R) was purchased from Bioss (Beijing, China). Rabbit anti-TGN46 pAbs (catalog no. ab50594), Alexa Fluor 555-goat anti-rabbit IgG pAbs (catalog no. ab150078), fluorescein isothiocyanate (FITC)-goat anti-pig IgG pAbs (catalog no. ab6911), and horseradish peroxidase (HRP)-goat anti-pig IgG pAbs (catalog no. ab6915) were purchased from Abcam (Cambridge, United Kingdom). Rabbit anti-PRRSV N pAbs (catalog no. GTX129270) and mouse anti-TSG101 MAb (catalog no. GTX70255) were purchased from GeneTex (San Antonio, TX). Alexa Fluor 647-mouse anti-TSG101 MAb (catalog no. NB200-112AF647) was purchased from Novus Biologicals (Littleton, CO). Mouse anti-dsRNA (J2) MAb (catalog no. 10010200) was purchased from Scicons (Budapest, Hungary). HRP-goat anti-mouse (catalog no. 115-035-003) or -rabbit (catalog no. 111-035-003) IgG pAbs were purchased from Jackson Immuno Research (West Grove, PA). Alexa Fluor 488-goat anti-mouse (catalog no. A-11029) or -rabbit (catalog no. A-11034) IgG pAbs, and Alexa Fluor 647-goat anti-mouse (catalog no. A-21236) or rabbit (catalog no. A-21245) IgG pAbs were purchased from Invitrogen (Carlsbad, CA). Mouse anti-PRRSV N and GP5 antibodies were kept in our laboratory (71). The PRRSV Nsp9 nanobody containing porcine IgG Fc (Nb6-pFc) was kindly provided by Qin Zhao from Northwest A&F University (72).

### Reagents.

Lipofectamine LTX with Plus reagent (catalog no. 15338030), Lipofectamine 2000 reagent (catalog no. 2066194), Lipofectamine RNAiMAX transfection reagent (catalog no. 13778150), NuPAGE LDS sample buffer (catalog no. NP0007), NuPAGE sample reducing agent (catalog no. NP0009), NativePAGE sample preparation kit (catalog no. BN2008), native PAGE 3%–12% Bis-Tris gels (catalog no. BN1001BOX), NuPAGE 12% Bis-Tris gel with 2D-well (catalog no. NP0346BOX), Dynabeads-protein G for immunoprecipitation (catalog no. 10004D), and dithiothreitol (DTT; catalog no. D1532) were purchased from Invitrogen. 4% paraformaldehyde (PFA; catalog no. P0099), 4′,6′-diamidino-2-phenylindole (DAPI; catalog no. C1005), radioimmunoprecipitation assay (RIPA) lysis buffer (catalog no. P0013B), NP-40 lysis buffer (catalog no. P0013F), Triton X-100 (catalog no. P0096), and proteinase K (catalog no. ST535) were purchased from Beyotime Biotechnology (Shanghai, China). Enhanced chemiluminescence (ECL) reagent (catalog no. P0013B) and 0.25% trypsin-EDTA solution (catalog no. C100C1) was purchased from NCM Biotechnology (Suzhou, China). Complete EDTA-free protease inhibitor cocktail (catalog no. 04693116001) and universal SYBR green master (catalog no. 04913914001) were purchased from Roche (Mannheim, Germany). A CellTiter 96 AQueous One solution cell proliferation kit (catalog no. G3582) was purchased from Promega (Madison, WI). PrimeScript RT master mix (catalog no. RR036B) and RNAiso Plus (catalog no. 9109) were purchased from TaKaRa (Dalian, China). *N*,*N*-Dimethylacrylamide (DMA; catalog no. 274135) was purchased from Sigma-Aldrich (St. Louis, MO). Anti-GFP magarose beads (catalog no. SM03805) were purchased from Smart-Lifesciences (Changzhou, China). Pierce anti-myc magnetic beads (catalog no. 88843) were purchased from Thermo Fisher Scientific (Waltham, MA). Minute ER enrichment kit (catalog no. ER-036) and Minute GA enrichment kit (catalog no. GO-037) were purchased from Invent Biotechnologies (Eden Prairie, MN).

### Expression vector construction and transfection.

The cDNA of TSG101 was amplified from MARC-145 cells and cloned into pcDNA3.1-myc/His_A and pEGFP plasmid to generate recombinant pcDNA3.1-myc/His-TSG101 and pEGFP-TSG101 expression vectors, respectively. The genes encoding PRRSV HN07-1 proteins were synthesized and cloned into pCAGGS-HA by Sangon Biotech (Shanghai, China). The expression vectors were transfected for 36 h with Lipofectamine 2000 or Lipofectamine LTX with Plus reagent according to the manufacturer’s instructions.

### RNA interference.

Three pairs of siRNAs targeting the ESCRT system and siRNA-NC were designed and synthesized by GenePharma (Shanghai, China). The cells were transfected with the indicated siRNAs for 36 h using Lipofectamine RNAiMAX according to the manufacturer’s instructions. The knockdown efficiencies were determined by RT-qPCR and IB. The transfected cells were applied for further assays. The indicated siRNAs are listed in Table 1.

**TABLE 1:** siRNAs used in this study

Target gene	Sequence (5′–3′)	
	Sense	Antisense
Monkey-HGS	GGCCAGAUCUUCUGUGGAATT	UUCCACAGAAGAUCUGGCCTT
Monkey-STAM1	GCAACAGUCAACCACCCUUTT	AAGGGUGGUUGACUGUUGCTT
Monkey-STAM2	GCAGGUACUUCAGAGUAUATT	UAUACUCUGAAGUACCUGCTT
Monkey-TSG101	CCUGAAGCAUGUACGUCUUTT	AAGACGUACAUGCUUCAGGTT
Monkey-VPS28	GGCUCAGAAAUCAGCACUATT	UAGUGCUGAUUUCUGAGCCTT
Monkey-VPS37A	GCCACUGUAGAAGAGCCAATT	UUGGCUCUUCUACAGUGGCTT
Monkey-VPS37B	UCCUCUGGAUUCCUUCAUUTT	AAUGAAGGAAUCCAGAGGATT
Monkey-VPS37C	CCGCUCAAACCUCUCAGAUTT	AUCUGAGAGGUUUGAGCGGTT
Monkey-MVB12A	GCCUGGAUACCUUCGAAUATT	UAUUCGAAGGUAUCCAGGCTT
Monkey-MVB12B	GCACAUCUCUCUAACACUUTT	AAGUGUUAGAGAGAUGUGCTT
Monkey-EAP20	GAGUCAAUCCAGAUUGUAUTT	AUACAAUCUGGAUUGACUCTT
Monkey-EAP30	UCUGAUAACUUUGGAGGAATT	UUCCUCCAAAGUUAUCAGATT
Monkey-EAP45	CCGUUCUCCUUUCCCAAAUTT	AUUUGGGAAAGGAGAACGGTT
Monkey-CHMP1A	GCAGAUCGCUGAGGAGAAUTT	AUUCUCCUCAGCGAUCUGCTT
Monkey-CHMP1B	GCUGGUGUGGUUAAGUCAATT	UUGACUUAACCACACCAGCTT
Monkey-CHMP2A	GGAGGAGAUGAUGAAUGAUTT	AUCAUUCAUCAUCUCCUCCTT
Monkey-CHMP2B	CCAAACAACUCGUGCAUCUTT	AGAUGCACGAGUUGUUUGGTT
Monkey-CHMP3	GCAAGCUGUAUGCCUCCAATT	UUGGAGGCAUACAGCUUGCTT
Monkey-CHMP4A	GCCUAUGGGCUUUGGAGAUTT	AUCUCCAAAGCCCAUAGGCTT
Monkey-CHMP4B	GGACAUUGCUGACCAGCAATT	UUGCUGGUCAGCAAUGUCCTT
Monkey-CHMP4C	CCAAAUGUGCCUUCCUCUUTT	AAGAGGAAGGCACAUUUGGTT
Monkey-CHMP5	CCACAGAUCCCUGCUUCAUTT	AUGAAGCAGGGAUCUGUGGTT
Monkey-CHMP6	GCGAUCACUCAGGAACAAATT	UUUGUUCCUGAGUGAUCGCTT
Monkey-CHMP7	GCAAUUGGAACCGACUCUATT	UAGAGUCGGUUCCAAUUGCTT
Monkey-IST1	GGAGAGAUACCUGAUUGAATT	UUCAAUCAGGUAUCUCUCCTT
Monkey-VPS4A	GGAGAUGACUUGGAUGGAUTT	AUCCAUCCAAGUCAUCUCCTT
Monkey-VPS4B	GGAUGUCCCUGGAGAUAAATT	UUUAUCUCCAGGGACAUCCTT
Monkey-VTA1	GCUCAGAAGUACUGCAAAUTT	AUUUGCAGUACUUCUGAGCTT
Monkey-ALIX	GCUUUCAAACAGUGUCAAUTT	AUUGACACUGUUUGAAAGCTT
Monkey-NEDD4	CCAAGAAGUCACAAAUCAATT	UUGAUUUGUGACUUCUUGGTT
Pig-TSG101-320	GUGCCUUAUAGAGGUAAUATT	UAUUACCUCUAUAAGGCACTT
Pig-TSG101-494	CACCCACAGUCAGAUUUGUTT	ACAAAUCUGACUGUGGGUGTT
Pig-TSG101-786	CCAGUAGAGAUGGCACAAUTT	AUUGUGCCAUCUCUACUGGTT
siRNA-NC	UUCUCCGAACGUGUCACGUTT	ACGUGACACGUUCGGAGAATT

### Cell viability assay.

The cells seeded in 96-well plates were transfected with siRNAs against ESCRT proteins or siNC for 60 h. The cell viability was measured using a CellTiter 96 AQueous One solution cell proliferation assay according to the manufacturer’s procedures. Briefly, the transfected cells were added with 20 μL of CellTiter 96 AQueous One solution reagent, followed by incubation for another 1 h to 4 h. The absorbance at 490 nm was recorded with a microplate reader (Fluostar Omega; BMG Labtech, Ortenberg, Germany).

### RT-qPCR.

Total RNAs were extracted using RNAiso Plus reagent and reversely transcribed into cDNAs using PrimeScript RT master mix kit. The cDNAs were amplified by PCR using universal SYBR green master on a RT-qPCR system (7500 Fast; Applied Biosystems, Foster City, CA). The relative mRNA abundance of different samples was evaluated using the 2^–ΔΔ^*^CT^* method (73), and GAPDH mRNA was set up as an endogenous control. The primers are listed in Table 2.

**TABLE 2:** Primers for RT-qPCR used in this study

Target gene	Sequence (5′–3′)	
	Sense	Antisense
Monkey-TSG101	TCCACCATACCAGGCAACG	AACCACTGGGATTGGGAGG
PRRSV-ORF7	AAACCAGTCCAGAGGCAAGG	GCAAACTAAACTCCACAGTGTAA
Monkey-GAPDH	TGACAACAGCCTCAAGATCG	GTCTTCTGGGTGGCAGTGAT
Pig-GAPDH	CCTTCCGTGTCCCTACTGCCAAC	GACGCCTGCTTCACCACCTTCT

### PRRSV titration assay.

The infected cells were subjected to three freeze-thaw cycles, centrifuged to eliminate cellular debris and the intracellular infectious virions were titrated by detecting TCID_50_ in MARC-145 cells. Instead, the infected cell supernatants were used to titrate extracellular infectious virions. Briefly, MARC-145 cells seeded in 96-well plates were infected with a 10-fold serial dilution of PRRSV at 37°C for 1 h. The viruses not entering into the cells were removed by washing with PBS. Each well was added with 100 μL fresh DMEM containing 2% FBS and then cultured for 3 to 5 days. The cells showing the expected cytopathic effect were counted daily, and the TCID_50_ value was calculated according to the method of Reed-Muench (74).

### IB.

The cells were rinsed with phosphate-buffered saline (PBS), lysed in RIPA lysis buffer containing protease inhibitor cocktail and incubated on ice for 30 min, followed by centrifugation at 12,000 × *g* at 4°C for 15 min. Cell lysates were normalized to equal amounts of GAPDH, loaded, and separated by 12% SDS-PAGE, followed by transferred onto 0.22-μm polyvinylidene fluoride (polyvinylidene difluoride) membranes (Merck Millipore, Darmstadt, Germany). The membranes were blocked in 5% skimmed milk at room temperature for 2 h, followed by incubation with the indicated primary antibodies at 4°C overnight. The membranes were washed three times with Tris-buffered saline containing 0.5% Tween 20 (TBST), followed by incubation with the secondary antibodies at room temperature for 2 h. After three washes with TBST, the immunoreactive bands were visualized with ECL reagent and imaged using a chemiluminescence imaging system (Fusion FX7; VILBER, Paris, France).

### IFA and confocal microscopy.

The monolayer cells were washed with cold-PBS, fixed with 4% PFA for 10 min, and permeabilized with 0.1% Triton X-100 in PBS at room temperature for 5 min. After three rinses with cold PBS, the cells were blocked with 5% bovine serum albumin (BSA) at room temperature for 1 h, followed by incubation with the appropriate primary antibodies at 4°C overnight. Subsequently, the cells were incubated with appropriate secondary antibodies, followed by staining cell nuclei with DAPI for an additional 5 min. The fluorescent images were acquired by a confocal laser scanning microscope (LSM700; Carl Zeiss AG, Oberkochen, Germany) with confocal laser scanning set up (20×, 40×, or 63×) and were representative as a single slice of a stack from three independent experiments (75). The numerical aperture (NA) of the 20× objective is 0.8, the NA of the 40× objective is 0.95, and the NA of the 63× objective is 1.4. The colocalization analyses were performed using the JaCoP plugin in ImageJ software according to previous research guidelines (44, 76, 77). Pearson’s correlation coefficient (>0.5) describes the correlation of the intensity distribution between channels. Manders’ overlap coefficient (>0.6) indicates an actual overlap of the signals and is considered to represent the true degree of colocalization. Manders’ colocalization coefficient describes the contribution of each selected channel to the pixels of interest, which represents the proportion of the colocalized fluorescence intensity of A protein with B protein to total fluorescence intensity of A protein. ImageJ software was also used to measure and analyze the single-channel fluorescence intensity (78, 79).

### FCM.

The PRRSV-infected MARC-145 cells were washed with PBS and digested with 0.25% trypsin-EDTA solution. After centrifugation at 2,000 × *g*, the cell pellets were fixed with 4% PFA, permeabilized with 0.1% Triton X-100, and blocked with 5% BSA. Then, the cells were incubated with mouse anti-PRRSV N MAb at room temperature for 2 h, followed by incubation with Alexa Fluor 647-goat anti-mouse IgG pAbs at room temperature for 1 h. Finally, the washed cells were resuspended in 300 μL of PBS, and the percentage of PRRSV-infected cells was analyzed using a flow cytometer (CytoFLEX; Beckman Coulter, Brea, CA).

### Attachment and internalization assay.

For PRRSV attachment assay, the siRNA-transfected cells were infected with PRRSV at an MOI of 10 and incubated at 4°C for 1 h. After adsorption, the unbound viruses were washed away with ice-cold PBS, and the relative level of cell-bound viral RNA was quantified by RT-qPCR (80). In parallel, PRRSV-bound cells were lysed for three freeze-thaw cycles and then subjected to a viral titration assay (81), or the cells seeded in glass dishes were infected with PRRSV (MOI = 10) and cultured at 4°C for 1 h. Then, the cell-bound virus was visualized using a mouse anti-GP5 antibody by confocal microscopy (82).

For PRRSV internalization assay, the cells were inoculated with PRRSV at an MOI of 10 at 4°C for 1 h. The unbound viruses were washed away with ice-cold PBS, and then the inoculated cells were transferred to 37°C for 1 h to allow viral internalization. The viruses not entering were removed with PBS containing proteinase K, and the entering viral RNA was analyzed by RT-qPCR or titration assay. Alternatively, the cells were transferred to 37°C for 30 min and then detected by confocal microscopy. For visualization of the colocalization of PRRSV with early endosomes, the cells were stained with a mouse anti-N MAb and rabbit anti-EEA1 pAbs, followed by an Alexa Fluor 488-goat anti-rabbit IgG antibody and an Alexa Fluor 647-goat anti-mouse IgG antibody.

### Transfection of infectious clone *in vitro*.

The HEK-293T cells grown in 12-well plates were transfected with siTSG101 or siNC for 24 h and then transfected with 2.5 μg of PRRSV infectious clone (HN07-GFP) using Lipofectamine 2000 reagent for another 36 h. The transfected cells were harvested for subsequent experiments.

### TEM.

The MARC-145 cells seeded in a 60-mm culture dish were transfected with siRNA duplexes (siNC or siTSG101). After 36 h, the transfected cells were infected with PRRSV at an MOI of 2 for 10 h. The cells were washed and centrifuged at 2,000 × *g* at 4°C for 2 min and then immobilized by using 2.5% glutaraldehyde fixative at room temperature for 30 min. The fixative was discarded, replaced with fresh fixative, and then transferred to 4°C overnight to continue the fixation. After three washes in double-distilled water, the fixed cells were dehydrated through a graded series of ethanol and finally embedded at 4°C in pure LR White resin (14381-UC; HaideBio, Beijing, China) using BEEM capsules (catalog no. 69920-00; Electron Microscopy Sciences, West Chester, PA). After polymerized with a low-temperature UV polymerizer (UVCC2515; Electron Microscopy China, Beijing, China), the resin blocks were cut into 70- to 80-nm thin slices on an ultramicrotome (UC7; Leica, Wetzlar, Germany), and the ultrathin sections were fished out onto the 150-mesh nickel grids with Formvar film. Finally, the sample grids were observed at 80 kV using TEM (Ht7800/Ht7700; Hitachi, Tokyo, Japan).

### GFP pulldown.

HEK-293T cells were coexpressed with EGFP-TSG101 and HA-tagged PRRSV proteins (HA-Nsp1α, -Nsp1β, -Nsp2, -Nsp4, -Nsp7, -Nsp9-12, and -N), respectively. The cells were washed with ice-cold PBS, lysed with NP-40 lysis buffer containing protease inhibitor cocktail, and incubated on ice for 30 min. The cell lysates were cleared by centrifugation at 12,000 × *g* for 15 min. The supernatants were incubated with GFP-magarose beads at 4°C overnight with rotation. Next, the samples were collected by using a magnetic rack and washed five times with TBS. The precipitated immunocomplexes were resuspended in 2× SDS loading buffer and then boiled at 95°C for 10 min. Finally, IB was conducted to detect the samples using mouse anti-HA MAb or mouse anti-GFP MAb.

### IP.

The MARC-145 cells infected with PRRSV or the HEK-293T cells coexpressed with myc/His-TSG101 and HA-N or HA-Nsp2 were washed with ice-cold PBS and lysed in NP-40 lysis buffer containing protease inhibitor cocktail. Cell lysates were cleared by centrifugation at 12,000 × *g* for 15 min. The endogenous TSG101 binding to N protein was tested according to the manufacturer’s instructions of the Dynabeads-protein G. Briefly, the supernatant was incubated with 0.5 μg of rabbit isotype IgG and 15 μL of Dynabeads-protein G at 4°C for 1 h with rotation. After the Dynabeads were removed by using a magnetic rack, the supernatants were incubated with rabbit anti-TSG101 antibody or rabbit isotype IgG at room temperature for 1 h. Then, the Dynabead-protein G mixture was added to the samples, followed by incubation for another 1 h. The precipitated immunocomplexes were collected using a magnetic rack, followed by PBS washes. Finally, the complexes were eluted with 2× SDS loading buffer and subjected to IB with the indicated antibodies. The exogenous TSG101 binding to HA-N or HA-Nsp2 in the HEK-293T cells was detected using Pierce anti-myc magnetic beads. The supernatants were incubated with the prewashed anti-myc magnetic beads at room temperature for 30 min. After being washed and eluted as described above, the potential associated proteins were detected by IB with mouse anti-myc MAb or mouse anti-HA MAb.

### Nucleocapsid oligomerization assays.

The assays were performed according to a previous literature (48). Briefly, the MARC-145 cells infected with PRRSV (MOI = 5) for 10 h were lysed using a NativePAGE sample preparation kit with 1% (vol/vol) *n*-dodecyl β-d-maltoside and 0.25% (vol/vol) G-250 sample additive under nonreduced conditions without heating according to the manufacturer’s instruction. Typically, 20 μL of the prepared sample was loaded onto NativePAGE 3%–12% Bis-Tris gels, and electrophoresis was performed at a constant voltage of 150 V. For two-dimensional resolution, individual lanes were cut out of the NativePAGE gel, reduced in 1× NuPAGE LDS sample buffer with 50 mM DTT at room temperature for 30 min, alkylated in 1 × NuPAGE LDS sample buffer with 50 mM DMA for 30 min, and finally quenched in 1× NuPAGE LDS sample buffer with 5 mM DTT and 20% ethanol for 15 min. Finally, the equilibrated gel strips were stacked perpendicularly above a NuPAGE 12% Bis-Tris gel with 2D-well and overlaid with 1 × NuPAGE LDS sample buffer. After electrophoresis, the nucleocapsid was detected by SDS-PAGE and IB.

### Enrichment of ER and GA proteins.

ER and GA were extracted with the Minute ER/GA enrichment kit according to the manufacturer’s instructions (83, 84). Briefly, for ER extraction, 3.0 × 10^7^ cells were collected, frozen at −80°C for 10 min, and then resuspended in 550 μL of buffer A. The cell suspension was transferred to a filter cartridge and centrifuged at 16,000 × *g* for 30 s. The pellets were resuspended and centrifuged at 2,000 × *g* for 5 min. All the supernatant was collected and centrifuged at 4°C, 8,000 × *g* for 10 min. After centrifugation, 400 μL of supernatant was transferred to a fresh tube and subsequently added with 40 μL of buffer B to the tube. The tube was incubated at 4°C for 30 min and centrifuged at 8,000 × *g* for 10 min. The pellets were collected and then resuspended with 400 μL of cold buffer A and 40 μL of buffer C. After incubation at room temperature for 15 min, the tube was centrifuged at 8,000 × *g* for 5 min. Subsequently, the 400 μL of supernatant and 400 μL of buffer D were added to a fresh tube, followed by incubation at 4°C for 20 min. After incubation, the tube was centrifuged at 10,000 × *g* for 10 min, and the precipitation material contained the enriched ER.

For GA extraction, after the cell suspension was collected, the tube was centrifuged at 4°C and 5,000 × *g* for 5 min without removing the filter. All of the supernatant was transferred to a fresh tube and centrifuged at 4°C and 16,000 × *g* for 30 min. After centrifugation, 400 μL of supernatant and 400 μL of buffer B were added to a fresh tube, followed by incubation on ice for 15 min. The tube was centrifuged at 8,000 × *g* for 5 min, and the precipitation material was resuspended in 200 μL of cold buffer A. After centrifugation at 8,000 × *g* for 5 min, the supernatant was transferred to a fresh tube, followed by incubation with 100 μL of cold buffer C on ice for 20 min. After incubation, the tube was centrifuged at 8,000 × *g* for 10 min, and the precipitation material contained the enriched GA.

### Statistical analysis.

Three replicates were included in each experiment, and each experiment was independently repeated at least three times. The experimental data are presented as group means ± the standard errors of means (SEM). All statistical analyses and calculations were performed with the unpaired two-tailed Student *t* test or one-way analysis of variance (ANOVA) using Prism 8.0 software (GraphPad, Inc., La Jolla, CA). Statistical significance in the figures is indicated by asterisks (*, *P* < 0.05; **, *P* < 0.01; ***, *P* < 0.001; ****, *P* < 0.0001; ns, not significant [*P* > 0.05]).
